# Estimation of Thalamocortical and Intracortical Network Models from Joint Thalamic Single-Electrode and Cortical Laminar-Electrode Recordings in the Rat Barrel System

**DOI:** 10.1371/journal.pcbi.1000328

**Published:** 2009-03-27

**Authors:** Patrick Blomquist, Anna Devor, Ulf G. Indahl, Istvan Ulbert, Gaute T. Einevoll, Anders M. Dale

**Affiliations:** 1 Department of Mathematical Sciences and Technology and Center for Integrative Genetics, Norwegian University of Life Sciences, Ås, Norway; 2 Athinoula A. Martinos Center, Massachusetts General Hospital, Charlestown, Massachusetts, United States of America; 3 Departments of Radiology and Neurosciences, University of California San Diego, La Jolla, California, United States of America; 4 Institute for Psychology of the Hungarian Academy of Sciences, Budapest, Hungary; 5 Peter Pazmany Catholic University, Department of Information Technology, Budapest, Hungary; University College London, United Kingdom

## Abstract

A new method is presented for extraction of population firing-rate models for both thalamocortical and intracortical signal transfer based on stimulus-evoked data from simultaneous thalamic single-electrode and cortical recordings using linear (laminar) multielectrodes in the rat barrel system. Time-dependent population firing rates for granular (layer 4), supragranular (layer 2/3), and infragranular (layer 5) populations in a barrel column and the thalamic population in the homologous barreloid are extracted from the high-frequency portion (multi-unit activity; MUA) of the recorded extracellular signals. These extracted firing rates are in turn used to identify population firing-rate models formulated as integral equations with exponentially decaying coupling kernels, allowing for straightforward transformation to the more common firing-rate formulation in terms of differential equations. Optimal model structures and model parameters are identified by minimizing the deviation between model firing rates and the experimentally extracted population firing rates. For the thalamocortical transfer, the experimental data favor a model with fast feedforward excitation from thalamus to the layer-4 laminar population combined with a slower inhibitory process due to feedforward and/or recurrent connections and mixed linear-parabolic activation functions. The extracted firing rates of the various cortical laminar populations are found to exhibit strong temporal correlations for the present experimental paradigm, and simple feedforward population firing-rate models combined with linear or mixed linear-parabolic activation function are found to provide excellent fits to the data. The identified thalamocortical and intracortical network models are thus found to be qualitatively very different. While the thalamocortical circuit is optimally stimulated by rapid changes in the thalamic firing rate, the intracortical circuits are low-pass and respond most strongly to slowly varying inputs from the cortical layer-4 population.

## Author summary

Many of the salient features of individual cortical neurons appear well understood, and several mathematical models describing their physiological properties have been developed. The present understanding of the highly interconnected cortical neural networks is much more limited. Lack of relevant experimental data has in general prevented the construction and rigorous testing of biologically realistic cortical network models. Here we present a new method for extracting such models. In particular we estimate specific mathematical models describing the sensory activation of thalamic and cortical neuronal populations in the rat whisker system from joint recordings of extracellular potentials in thalamus and cortex. The mathematical models are formulated in terms of average firing rates of a thalamic population and a set of laminarly organized cortical populations, the latter extracted from data from linear (laminar) multielectrodes inserted perpendicularly through cortex. The identified models describing the signal processing from thalamus to cortex are found to be qualitatively very different from the models describing the processing between cortical populations; while the thalamocortical circuit is optimally stimulated by rapid changes in the thalamic firing rate, the intracortical circuits respond most strongly to slowly varying inputs.

## Introduction

Following pioneering work in the 1970s by, e.g., Wilson and Cowan [1] and Amari [2] a substantial effort has been put into the investigation of neural network models, particularly in the form of *firing-rate* or *neural field* models [3]. Some firing-rate network models, in particular for the early visual system ([4], Ch.2), have been developed to account for particular physiological data. However, for strongly interconnected cortical networks, few mechanistic network models directly accounting for specific neurobiological data have been identified. Instead most work has been done on generic network models and has focused on the investigation of generic features, such as the generation and stability of localized bumps, oscillatory patterns, traveling waves and pulses and other coherent structures, for reviews see Ermentrout [5] or Coombes [6].

We here (1) present a new method for identification of *specific* population firing-rate network models from extracellular recordings, (2) apply the method to extract network models for thalamocortical and intracortical signal processing based on stimulus-evoked data from simultaneous single-electrode and multielectrode extracellular recordings in the rat somatosensory (barrel) system, and (3) analyze and interpret the identified firing-rate models using techniques from dynamical systems analysis. Our study reveals large differences in the transfer function between thalamus (VPM) and layer 4 of the barrel column, compared to that between cortical layers, and thus sheds direct light on how whisker stimuli is encoded in population firing-activity in the somatosensory system.

The derivation of biologically realistic, cortical neural-network models has generally been hampered by the lack of relevant experimental data to constrain and test the models. Single electrodes can generally only measure the firing activity of individual neurons, not the joint activity of populations of cells typically predicted by population firing-rate models. Kyriazi and Simons [7] and Pinto et al. [8],[9] thus developed models for the somatosensory thalamocortical signal transformation based on pooled data from single-unit recordings from numerous animals. By contrast, multielectrode arrays provide a convenient and powerful technology for obtaining simultaneous recordings from all layers of the cerebral cortex, at one or more cortical locations [10]. The signal at each low-impedance electrode contact represents a weighted sum of the potential generated by synaptic currents and action potentials of neurons within a radius of a few hundred micrometers of the contact, where the weighting factors depend on the shape and position of the neurons, as well as the electrical properties of the conductive medium [11]–[13].

In the present paper we describe a new method for extraction of population firing-rate models for both thalamocortical and intracortical transfer on the basis of data from simultaneous thalamic single-electrode and cortical recordings using linear (laminar) multielectrodes in the rat barrel system. With so called *laminar population analysis (LPA)* Einevoll et al. [11] jointly modeled the low-frequency (*local field potentials; LFP*) and high-frequency (*multi-unit activity; MUA*) parts of such stimulus-evoked laminar electrode data to estimate (1) the laminar organization of cortical populations in a barrel column, (2) time-dependent population firing rates, and (3) the LFP signatures following firing in a particular population. These ‘postfiring’ population LFP signatures were further used to estimate the synaptic connection patterns between the various populations using both *current source density (CSD)* estimation techniques and a new LFP template-fitting technique [11].

Here we use the stimulus-evoked time-dependent firing rates for the cortical populations estimated using LPA, in combination with single-electrode recordings of the firing activity in the homologous barreloid in VPM, to identify population firing-rate *models*. The models are formulated as nonlinear Volterra integral equations with exponentially decaying coupling kernels allowing for a mapping of the systems to sets of differential equations, the more common mathematical representation of firing-rate models [5],[14].

The population responses were found to increase monotonically both with increasing amplitude and velocity of the whisker flick [11],[15],[16]. A stimulus set varying both the whisker-flicking amplitude and the rise time was found to provide a rich variety of thalamic and cortical responses and thus to be well suited for distinguishing between candidate models. The optimal model structure and corresponding model parameters are estimated by minimizing the mean-square deviation between the population firing rates predicted by the models and the experimentally extracted population firing rates.

A first focus is on the estimation of mathematical models for the signal transfer between thalamus (VPM) and the layer-4 population, the population receiving the dominant thalamic input. For this thalamocortical transfer our experimental data favors a model with (1) fast feedforward excitation, (2) a slower predominantly inhibitory process mediated by a combination of recurrent (within layer 4) and feedforward interactions (from thalamus), and (3) a mixed linear-parabolic activation function. The identified thalamocortical circuits are seen to have a band-pass property, and in the frequency domain the largest responses for the layer-4 population is obtained for thalamic firing rates with frequencies around twenty Hz.

Very different population firing-rate models are identified for the intracortical circuits, i.e., the spread of population activity from layer 4 to supragranular (layer 2/3) and infragranular (layer 5) layers. For the present experimental paradigm the extracted firing rates of the various cortical laminar populations are found to exhibit strong temporal correlations and simple feedforward models with linear or mixed linear-parabolic activation function are found to account excellently for the data. The functional properties of the identified thalamocortical and intracortical network models are thus qualitatively very different: while the thalamocortical circuit is optimally stimulated by rapid changes in the thalamic firing rate, the intracortical circuits are low-pass and respond strongest to slowly varying inputs.

Preliminary results from this project were presented earlier in poster format [17].

## Results

Here we illustrate our approach by first showing results from one of the six experimental data sets considered, and next show the thalamic and cortical laminar population responses extracted from these experimental data. Further, we outline the general form of the neural population models we explore to account for these population firing. We then go on to test specific candidate *thalamocortical* models against the experimentally observed population responses in layer 4 using the experimental thalamic response as driving input to the model. The identified thalamocortical models found by minimizing the deviation between model predictions and experimental population firing rates are further analyzed and explored using tools from dynamical system analysis. Finally, we correspondingly examine how the experimentally measured laminar population responses in layer 2/3 and layer 5, respectively, can be explained by *intracortical* network models with the experimental layer-4 population responses as input.

### Experimental data and extracted population firing rates

**Figure 1:**
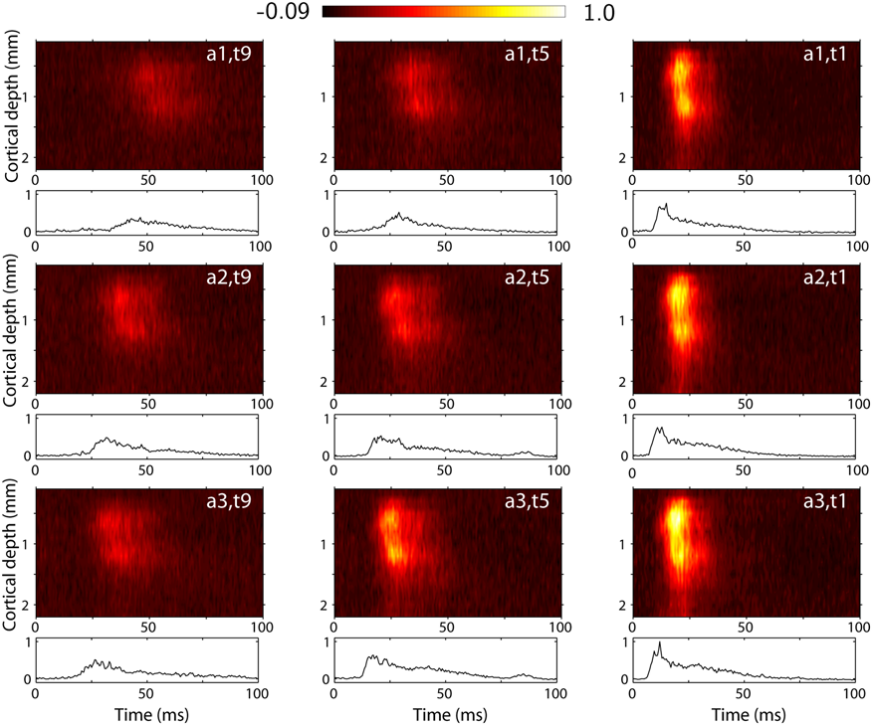
Example experimental data. Trial-averaged experimental data for experiment 1 for 9 out of 27 applied stimulus conditions for three different amplitudes (rows) and three different rise times (columns). Contour plots show depth profile of MUA as recorded by the laminar electrode, normalized to its largest value over the 22 electrode traces and 27 stimulus condition. Line plots below show the corresponding thalamic MUA recorded by a single electrode, normalized to the largest value over the 27 stimulus conditions.

In Figure 1 we show trial-averaged *multi-unit activity (MUA)* data for one of six experiments, labeled ‘experiment 1’, from stimulus onset, i.e., onset of whisker-flick, until 100 ms after. This is the time window of data used in the further analysis. Color plots depict the laminar-electrode recordings while the line plots below show the corresponding trial-averaged thalamic recordings. Results for 9 of 27 stimulus conditions are shown, corresponding to three different stimulus rise times 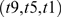 for three different stimulus amplitudes 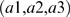. Neurons in the barrel cortex are sensitive to both stimulus amplitude and stimulus velocity (and thus stimulus rise time) [11],[15],[16], and we found that a set of stimuli varying both the amplitude and rise time provide a rich variety of responses suitable for the present study.

**Figure 2:**
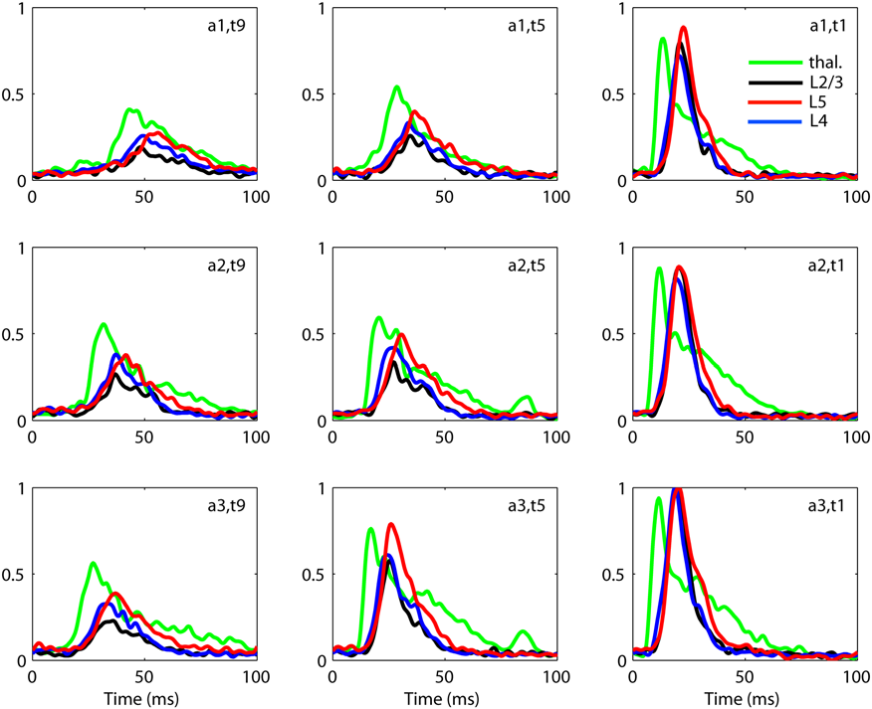
Example extracted population firing rates. Extracted population firing rates for experiment 1 for 9 out of 27 applied stimulus conditions. Green: thalamus. Black: layer 2/3. Blue: layer 4. Red: layer 5. Note that the extracted firing rates are normalized so that the maximum response over all stimulus conditions is unity. For layer 2/3, layer 4 and layer 5 this occurs for the stimulus 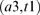 shown in the figure. For the thalamic rate it occurs for stimulus 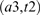, not shown in the figure.

Fig. 2 shows the estimated thalamic 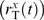 and cortical 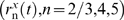 population firing rates extracted from the data in Fig. 1 (procedure described in Materials and Methods). The estimated thalamic population firing rate is essentially just a low-pass filtered version of the thalamic MUA. The cortical population firing rates have been estimated by a method from the recently developed *laminar population analysis (LPA)* [11] where the MUA from the full set of electrode contacts on the laminar electrode is modeled as a sum over contributions from a set of laminar populations, cf. Eqs. (16–17). In the present case four cortical populations are assumed in this firing-rate extraction scheme [11], but the figure only shows results for the three cortical populations considered in this study, namely the layer 2/3 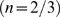, layer 4 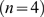, and layer 5 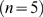 populations. A deeper laminar population was also identified, but it was left out of the present analysis since predictions related to the postsynaptic LFP-profiles from this particular population were found to vary between different experiments, thus questioning its proper identification [11].

### Form of thalamocortical model

We first focus on the signal transfer between thalamus and the dominant input layer of the barrel column, namely layer 4. Specifically we seek a mathematical model predicting the layer-4 population firing rate 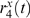 for all 27 stimulus conditions (of which nine are depicted in Fig. 2) given the corresponding thalamic population firing 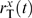 as model input.

The stimulus-evoked dynamics of the excitatory and inhibitory neurons in layer 4 of the barrel column is complex. The excitatory neurons receive both feedforward excitation from thalamic neurons and recurrent excitation from other layer 4 excitatory cells [9], [18]–[20]. In addition, the excitatory cells are inhibited by layer-4 interneurons [21]. These interneurons in turn receive feedforward excitation from thalamus [22]–[24] and recurrent inputs from other layer-4 neurons [9],[21].

For mathematical convenience and conceptual simplicity we choose to formulate the population firing-rate model as Volterra integral equations [5],[14]. In our so called *full thalamocortical* model where all the abovementioned feedforward and recurrent connections are included, we then have
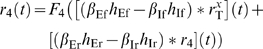
(1)


Here the 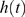 are *temporal coupling kernels*, and 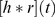 is a *temporal convolution* between the temporal kernel 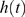 and the population firing rate 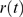 (Eq. 19). We here assume exponentially decaying temporal coupling kernels (Eq. 20) which allows for a mapping of the integral equation to a set of differential equations [5],[14].

**Figure 3:**
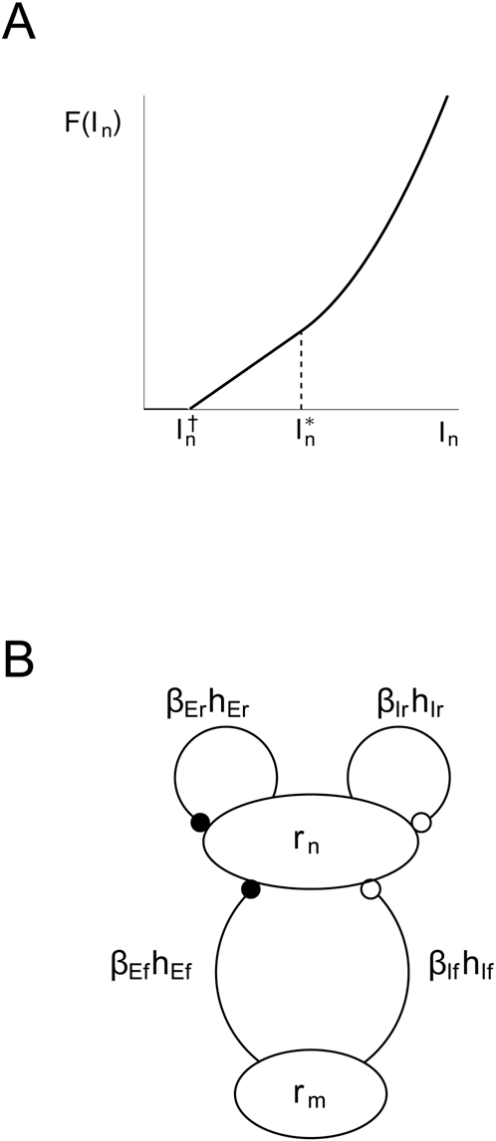
Model structure. (A) Illustration of form of activation function 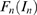 in Eq. (21). (B) Illustration of structure of population firing-rate models with feedforward and recurrent terms in Eqs. (1) and (10). Filled and open dots represent excitatory and inhibitory connections, respectively.

The four terms within the large parentheses on the right hand side of Eq. (1) can be interpreted as four contributions to the input current entering the somas of neurons in the layer-4 population. The first and second terms correspond to *feedforward* excitation 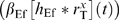 and inhibition 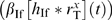 from the thalamus, respectively. The coupling kernel 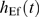 can be interpreted as the weight of the excitatory contribution to the present current input to the layer-4 population due to firing that has occurred in the thalamic population a time 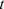 in the past. Likewise, the third and fourth terms correspond to *recurrent* excitation 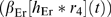 and inhibition 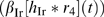 from cortical layer-4 neurons. The model structure is illustrated in Fig. 3B. Note that corticothalamic feedback [25] is not explicitly modeled as this effect will only change the thalamic firing rate. Since we do not model the thalamic firing rates and instead use the experimentally extracted 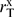 as input to the model, any effect of cortical feedback affecting the thalamic firing rate will be included automatically.

The function 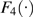 in Eq. (1) is an *activation function* that converts the net input current to population firing rate 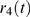. This activation function is often modeled as a sigmoidal function [4],[8], but here we found that a simpler four-parameter threshold-type function with a linear and/or parabolic activation above threshold provided a better fit to the experimental data, cf. Eq. (21). The form of this activation function is illustrated in Fig. 3A. Such a threshold-type activation function has also been seen experimentally when studying firing characteristics of excitatory neurons in the barrel column [26].

The temporal coupling kernel 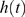 is described by two parameters, a time constant 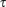 and a time-delay parameter 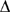, cf. Eq. (20). To reduce the number of parameters in the model in Eq. (1), the time delays in the recurrent temporal kernels are set to zero, i.e., 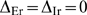. Further, one of the weight parameters 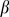 can be fixed without loss of generality. We therefore set 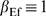. Nevertheless, this full thalamocortical model encompasses 13 parameters (4 for the activation function, 3 weights, 6 specifying temporal kernels). This is a sizable number of adjustable model parameters given the variability of the experimental data, and when comparing with our experimental data it was observed that in particular the feedforward and recurrent inhibition terms had strongly overlapping effects on the model predictions for the layer-4 firing rate so that their individual contributions were difficult to assess. We thus chose to mainly investigate reduced versions of the full thalamocortical model in Eq. (1) where either (1) only the recurrent inhibition term (‘recurrent’ model) or (2) only the feedforward inhibition term (‘feedforward’ model) was kept, respectively.

### Recurrent thalamocortical model

We first consider the so called *recurrent* thalamocortical model where the feedforward inhibitory term in the full model in Eq. (1) has been omitted, i.e.,
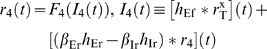
(2)


This model has a special significance in that it maps (by use of the linear chain trick [14]) to a set of differential equations that are structurally similar to the firing-rate model suggested by Pinto et al. [8],[9], cf. Eqs. (28–29) in Materials and Methods.

The model was fitted to the experimentally extracted population firing rate 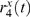 (for all 27 stimulus conditions simultaneously) by minimizing the mean square deviation between model and experimental results (Eq. 22) using the optimization method described in Materials and Methods.

#### Fits to recurrent model

**Figure 4:**
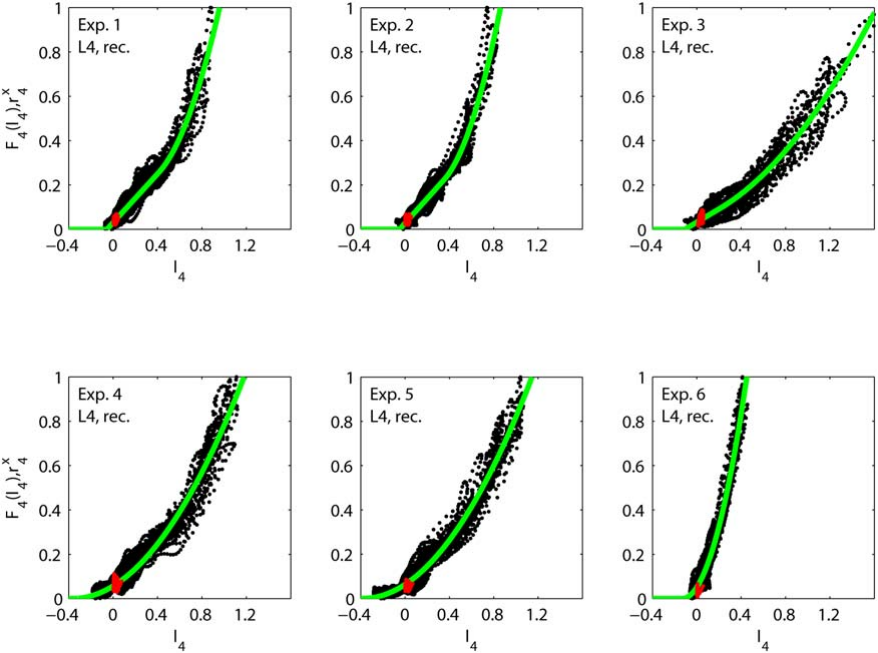
Fits of recurrent thalamocortical model. Illustration of fits of recurrent thalamocortical model in Eq. (2) to data from experiments 1–6. Each black dot corresponds to the experimentally measured layer-4 firing rate 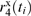 at a specific time point 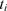 plotted against the model value of 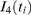. The red dots are corresponding experimental data points taken from the first 5 ms after stimulus onset (for all 27 stimuli). These data points show the activity prior to any stimulus-evoked thalamic or cortical firing and correspond to background activity. The solid green curve corresponds to the fitted model activation function 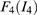.

As illustrated in Fig. 4 the recurrent model successfully accounts for the experimental observations in all six experiments considered. In the panels each dot corresponds to an experimentally measured layer-4 firing rate 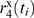 at a particular time 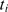 plotted against the value 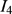 predicted by the fitted model at the same time 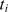. If the model was perfect and the data noiseless, these points should all lie on the solid line corresponding to the fitted value of the activation function 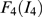. A small spread is generally observed in Fig. 4, and this is reflected by the low error value, i.e., 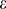 in Eq. (22), of the best fits. As seen is Table 1 the errors are all less than 6%. In Table 1 we also list the fitted parameter values for the six experiments.

**Table 1:** Fitted model parameters for recurrent thalamocortical model.

	Exp. 1	Exp. 2	Exp. 3
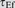 (ms)	3.7	5.7	3.3
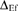 (ms)	2.5	2.0	4.5
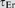 (ms)	9.3	8.0	1.3
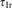 (ms)	13.7	14.8	30.6±0.7
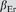	4.27±0.03	2.93±0.16	1.26±0.03
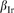	4.81±0.03	3.85±0.16	1.48±0.02
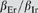	0.89	0.76±0.01	0.85±0.02
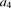	0.55	0.56	0.30
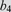	1.48	1.88±0.01	0.26±0.02
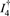	−0.06	−0.05	−0.11
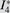	0.41	0.35	0.21
error 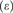	0.0522	0.0430	0.0586

Resulting optimized parameters for the recurrent thalamocortical network model (Eq. 2) incorporating feedforward excitation and recurrent excitation and inhibition. The listed parameter values correspond to the mean of fitted parameter values from 25 selected models giving essentially the same error (see Materials and Methods). The standard deviations are only listed if they exceed the last digit of the mean. Note that the value of 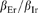 is included to illustrate the relative constancy of the ratio of the fitted values for 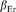 and 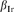.

#### Fitted model parameters for recurrent model

Experiments 1–3 correspond to the stimulus paradigm with 3 different amplitudes and 9 different rise times, while experiments 4–6 correspond to 27 different amplitudes but a single fixed rise time. As seen, for example, in Fig. 2 the variation in the rise time affects the thalamic and cortical responses more than variation in the stimulus amplitude. Thus experiments 1–3 exhibit a greater variation in the responses and thus a richer data set for the models to be tested against. In fact, for experiments 4–6 only a parabolic part of the activation function 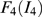 could be seen in the data, and the linear part of the activation function in Eq. (21) was omitted from the model.

The choice of stimuli set also appears to affect the fitted parameter values somewhat as indicated by comparing the fitted parameter values for experiments 1, 2, 4 and 5, all carried out using the same preparation in the same rat. As seen in Table 1 the fitted parameter values of experiment 1 and 2 are very similar, likewise for experiment 4 and 5. The parameter values for experiments 1 and 2 also compare well with the values for experiments 4 and 5, but less so. In the following we will focus mostly on experiments 1–3 where the 3×9 stimulus paradigm is used, and a more varied set of responses are obtained.

For experiments 1 and 2 the fitted feedforward time constants 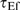 are between 3 and 6 milliseconds and the feedforward delay 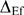 between 2 and 3 milliseconds (see Table 1). The recurrent time constants are significantly longer: between 8 and 10 milliseconds for the recurrent excitation 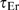 and between 13 and 15 milliseconds for the recurrent inhibition 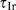. For all six experiments we observe the recurrent inhibition to have the longest time constant. In experiments 3 and 6 the fitted time constant of the feedforward excitation is seen to be longer than for the recurrent excitation.

In all experiments the weight of the recurrent inhibition 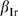 was found to be larger than the weight of the recurrent excitation 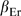. However, the values of 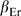 and 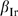 are seen to vary significantly between the experiments. This partially reflects that when the recurrent terms dominate the feedforward term, large values of the recurrent weights can be compensated by making the activation function less steep, and vice versa. However, the ratio 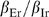 is found to be rather constant, typically between 0.75 and 0.9 (with experiment 6 as the only exception). Thus while excitation may dominate at short time scales due to the shorter excitatory time constants, inhibition dominates at longer time scales.

The positive correlation between 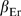 and 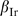 was confirmed by direct inspection of the correlation matrices for the ensembles of fitted model parameters obtained during the numerical optimization procedure (Materials and Methods). It was also seen by inspection of the eigenvectors of the most ‘sloppy’ direction of the Levenberg-Marquardt Hessian 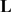 (Eq. 23), i.e., the component with the smallest eigenvalue. A ‘sloppy’ direction of 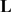 means that the fitting error 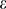 will change little when moving along this direction in parameter space [27]. A positive correlation between 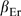 and 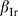 would imply that the fitting error will change little when both parameters are increased (or decreased) simultaneously. Indeed, the eigenvector of the most ‘sloppy’ eigenvalue revealed exactly this property.

Further inspection revealed that 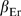 is negatively correlated with the slope of the activation function (parameters 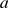 and 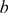 in Eq. (21)). This is as expected since a decrease in the recurrent excitatory weight 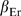 can be compensated for by an increase in the activation-function slope. Moreover, it was observed that 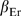 (and 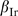) is positively correlated to 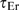 and negatively correlated to 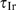.

#### Phase-plane analysis of recurrent model

The identified recurrent thalamocortical models on integral form can be mapped to a corresponding set of two differential firing-rate equations using the ‘linear-chain trick’ [5],[14] as described in Materials and Methods, cf. Eqs. (25–29). This is very useful since it allows for the use of standard techniques from dynamical systems analysis to investigate the identified models.

With slightly redefined auxiliary variables compared to Eqs. (25–27) in Materials and Methods, that is,
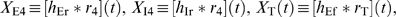
(3)the recurrent thalamocortical model can be reformulated as (cf. Eqs. 28–29)
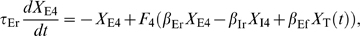
(4)

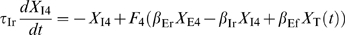
(5)


This equation set resembles the model derived using ‘semirigorous’ techniques by Pinto et al. in [8], and further explored in [9], for populations of interconnected layer-4 excitatory and inhibitory barrel neurons receiving stimulus-evoked thalamic input.

**Figure 5:**
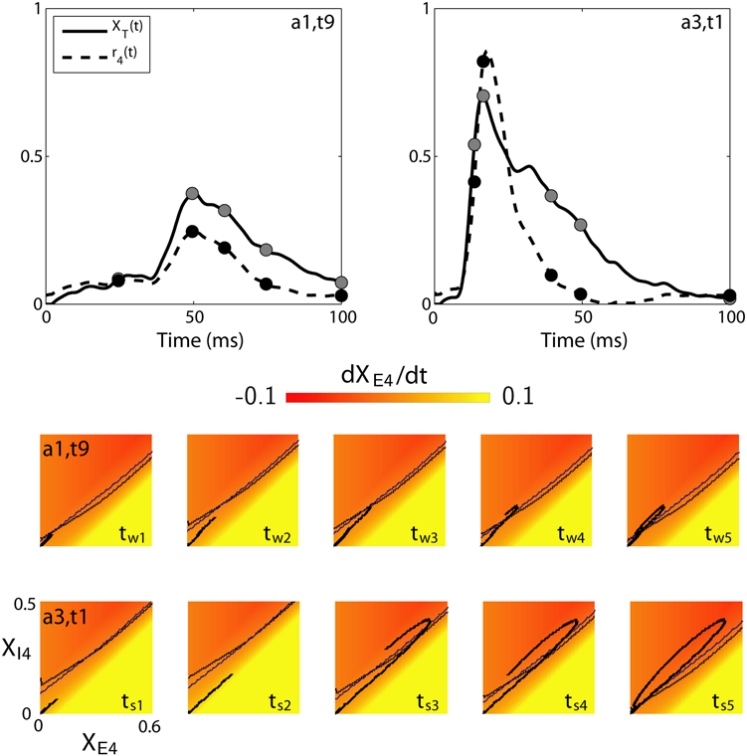
Phase-plane analysis of recurrent thalamocortical model for experiment 1. Phase-plane analysis of recurrent thalamocortical model in Eqs. (4–5) for fitted model parameters for experiment 1, cf. Table 1. Upper panels show the synaptic drive from thalamus 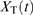 and the firing rate 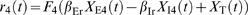 for the weakest (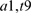; left) and strongest (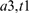; right) stimuli. The filled dots in the two upper panels show the points in time, 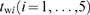 for 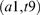 and 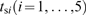 for 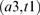 at which the network states are shown in the two lower panels. In each of the phase plots (five for each of the stimuli) the *instantaneous* excitatory (black) and inhibitory (blue) nullclines are shown, i.e., the nullclines obtained with a constant thalamic synaptic drive equal to the instantaneous value 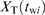 for 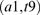 and 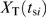 for 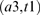. The network's activity is indicated by the black response curve, where the head of the curve marks the network's current state, and the tail represents prior states.

The formulation in terms of two dynamical variables 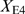 and 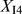, with 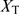 only providing an external input, allows for visualization of the circuit behavior using phase-plane analysis. In analogy with Pinto et al. [9] we show in Fig. 5 the layer-4 model response for the weakest 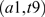 and strongest 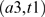 stimuli for experiment 1. In the five phase plots for the two stimuli considered the *instantaneous* excitatory and inhibitory nullclines at five different times (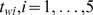 for weak stimulus; 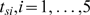 for strong stimulus) are shown. These nullclines are obtained assuming a constant thalamic synaptic drive equal to the instantaneous values 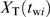 or 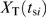. Below the instantaneous excitatory (inhibitory) nullcline 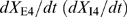 is negative, and the color code illustrates the magnitude of 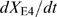.

As seen in Fig. 5 the network model for the strong stimulus 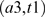 makes a significantly more extended excursion in the 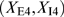 phase plane than the weaker stimulus 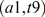. This can be readily understood by investigation of the phase-plane plots for the times shortly after onset. For the strong stimulus the trajectory is seen to be in a region with a large 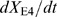 at, for example, the times 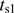 and 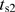 resulting in a rapid growth and large maximum value of 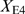. For these short times the fast feedforward and recurrent excitation dominates the slower inhibition. For the weak stimulus, however, the trajectory remains in regions with small values of 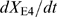 (i.e., without dominant excitation). As a consequence we observe that while the difference in maximum amplitude of the thalamic synaptic drives between the strong and weak stimuli is seen in the upper panels of Fig. 5 to be less than a factor two, the difference in the maximum layer-4 firing rate is more than a factor three. Thus the amplitude of the thalamic population response does not explain the layer-4 response alone; the rise time of the thalamic response is also important [9].

The qualitative conclusions from Fig. 5 is in agreement with the corresponding analysis by Pinto et al. [9]. This is an interesting result in itself since our model and the model of Pinto et al. [9] are derived in very different ways. Here the parameters are extracted from a single experiment measuring population activity directly. By contrast, in the study by Pinto et al. the model and its parameters were chosen to qualitatively reproduce pooled barrel population responses, i.e., single-unit poststimulus time histograms (PSTHs), recorded from numerous animals [8],[9]. Further, the thalamic responses used to generate their phase-plots analogous to Fig. 5 were modeled with a simplified triangular temporal rate profile with varying rise times [9], not extracted from experimental data like here.

Due to differences between our model (Eqs. 4–5) and the model of Pinto et al. [9] a direct comparison between the applied model parameters is generally not possible. However, the time constants can be compared directly: their choice of *τ*_Er_ = 5 ms and *τ*_Ir_ = 15 ms is qualitatively similar to what was extracted from our experiments 1 and 2, i.e., *τ*_Er_ = 8–10 ms and *τ*_Ir_ = 13–15 ms. Their choice of weight parameters appears to differ more from our results: for example, they set the ratio 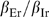 to be between 1.7 and 2.3, i.e., stronger recurrent excitation than recurrent inhibition. For experiments 1–3 we see in Table 1 that the ratio is found to be between 0.75 and 0.9, i.e., the recurrent inhibition has a larger weight than recurrent excitation. For the *tension*, i.e., the ratio of recurrent excitation over the excitation from thalamus 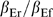 [8], they chose a value of about 0.9. For our experiments this ratio showed a significant variation, varying from 1.3 to 4.3 for experiments 1–3.

#### Stability of background states for recurrent model

On differential form our population-firing rate model is readily available for stability analysis. Due to the recurrent excitatory term the recurrent thalamocortical model can in principle have unstable equilibrium points, but the identified models must clearly exhibit stable background states when only the stationary thalamic input (corresponding to the absence of whisking) is present. For experiments 1–3 the background state (cf. red dots in Fig. 4) is found to be on the linear flank of the activation function, and the stability of these background states can be demonstrated on the basis of the fitted model parameters 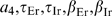 listed in Table 1. A full stability analysis for the recurrent thalamocortical model is given in Materials and Methods (Eqs. 30–35), and the criteria for stability of the background state is found to be
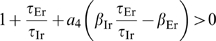
(6)

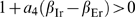
(7)


Insertion of the fitted numerical parameter values into the inequalities Eqs. (6–7) demonstrates that both conditions are fulfilled and that the background state is indeed stable. Inspection of Eqs. (6–7) further shows that instability is promoted by an increased weight of the recurrent excitation 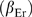, an increased slope of the activation function 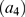, or an increased time constant of the recurrent inhibition 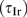 [28]. For example, for experiment 1 the background state becomes unstable if 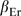 is increased by a factor 1.5, 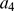 by a factor 3.2, or 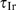 by a factor 1.9.

### Inclusion of feedforward inhibition in thalamocortical model fits

We further investigated whether the full thalamocortical model in Eq. (1), i.e., the recurrent model amended with the feedforward inhibition term 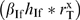, gives an improved fit compared to the recurrent model (Eq. 2) alone. Numerical investigations showed that the improvement, i.e., reduction of the error 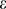, was marginal: for experiments 1–3 the reduction in error was in all cases less than 0.001. Thus the recurrent inhibitory term alone seems to be sufficient to account well for the present experimental data.

**Figure 6:**
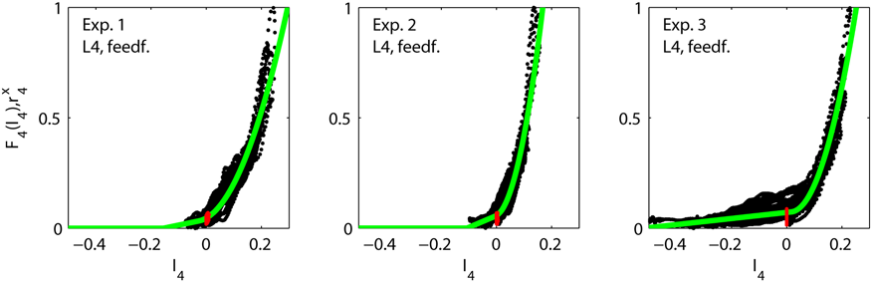
Fits of feedforward thalamocortical model. Illustration of fits of feedforward thalamocortical model (Eq. 8) to data from experiments 1–3. Each dot corresponds to the experimentally measured layer-4 firing rate 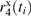 at a specific time point 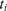 plotted against the model value of 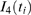. The red dots are corresponding experimental data points taken from the first 5 ms after stimulus onset (for all 27 stimuli). These data points show the activity prior to any stimulus-evoked thalamic or cortical firing and represent background activity. The solid green curve corresponds to the fitted model activation function 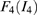.

We then investigated whether a pure *feedforward* model, i.e., a model with feedforward excitation and inhibition only, could account for the thalamocortical data. This model reads
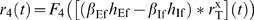
(8)and in Table 2 and Fig. 6 we show the results of the optimization.

**Table 2:** Fitted model parameters for feedforward thalamocortical model.

	Exp. 1	Exp. 2	Exp. 3
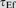 (ms)	8.4	9.8	9.3
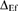 (ms)	2.5	3.5	5.0
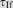 (ms)	20.5±0.3	18.2±0.1	100^*^
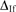 (ms)	2.5	3.5	5.0
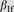	0.94	1.06	3.61
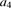	0.28±0.02	0.54±0.01	0.14
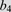	8.9±0.2	29.5±1.2	16.2±0.3
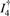	−0.15±0.01	−0.11	−0.52
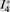	−0.03	−0.00	0.02
error 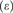	0.0636	0.0691	0.0706

Resulting optimized parameters for the feedforward thalamocortical network model (Eq. 8) incorporating feedforward excitation and inhibition. The listed parameter values correspond to the mean of fitted parameter values from 25 selected models giving essentially the same error (see Materials and Methods). The standard deviations are only listed if they exceed the last digit of the mean. Note that for experiments 4–6 the parameter 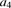 was fixed to zero in the optimization procedure. Also, for experiment 3 the listed value 100 ms for 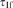 corresponds to the maximum allowed value for this parameter.

### Comparison of fits to recurrent and feedforward thalamocortical models

For experiments 1–3, which exhibit the largest variation of responses, the fits of the experimental data for the feedforward model are poorer than for the corresponding fits of the recurrent model. While the errors 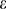 for the recurrent model are seen in Table 1 to be 0.052, 0.043 and 0.059 for experiments 1, 2 and 3, respectively, the corresponding errors for the purely feedforward model are seen in Table 2 to be 0.064, 0.069 and 0.071, i.e., relative increases in errors of 23%, 60% and 20%, respectively. However, for experiments 4–6 where only the stimulus amplitude and not the rise time is varied, there is no such clear preference, i.e., lower fitting errors, for the recurrent model.

**Figure 7:**
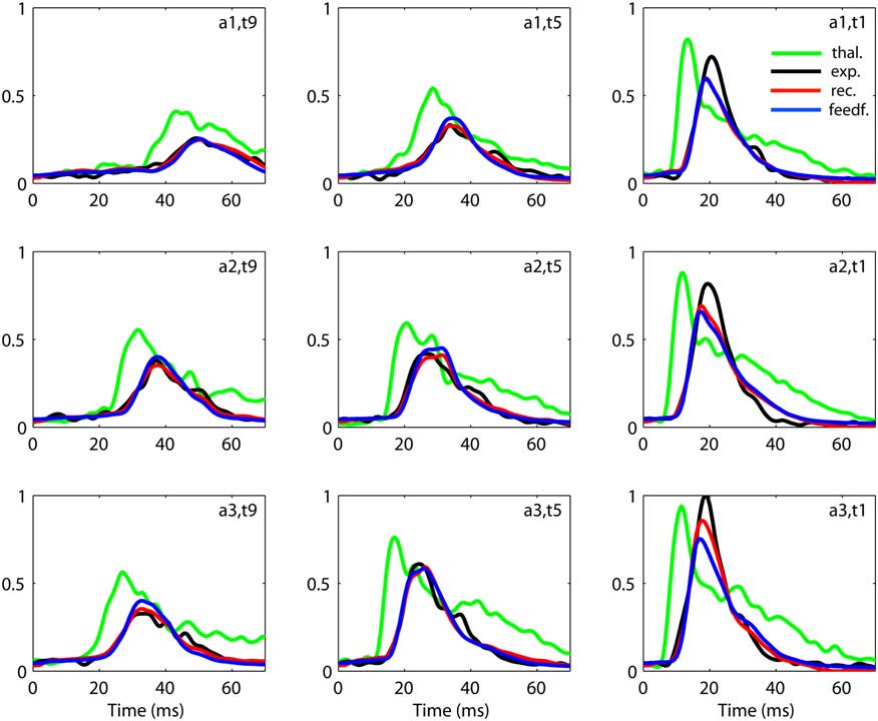
Comparison of thalamocortical model fits. Comparison of fits of the recurrent and feedforward thalamocortical models with experimental data set 1 for the same 9 (of 27) stimulus conditions considered in Fig. 2. The green line corresponds to the thalamic input firing rate 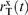, the black line to the experimentally extracted layer-4 firing rate 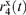, while the red and blue lines correspond to the best fits of the recurrent (Eq. 2) and feedforward (Eq. 8) models for the layer-4 firing rate 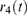, respectively. Time zero corresponds to stimulus onset.

In Fig. 7 we compare the best fits of the recurrent (Eq. 2) and feedforward (Eq. 8) thalamocortical models with the experimentally extracted layer-4 firing rate for experiment 1 for the 9 stimulus conditions depicted in Fig. 2. Both models underestimate the peak values for the layer-4 firing rate for the shortest rise time 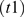 giving the largest responses. For the stimulus 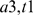 giving the overall largest layer-4 response, the fitted recurrent model both follows the rise and the ensuing fall of the response peak better than the feedforward model, but this difference is less pronounced for the other stimuli providing the larger responses. A detailed investigation of the fits for individual stimulus conditions in fact shows that the fitted recurrent model typically is closer to the experimental curves than the feedforward model for the entire set of stimulus conditions (data not shown).

The fitted activation functions for the recurrent and feedforward models for experiments 1 to 3 are quite different (cf. Figs. 4 and 6). In particular, for experiments 1 and 2 the activation functions for recurrent models have pronounced linear flanks which cover about half the dynamic range of 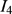. In contrast, the activation functions of the feedforward models for these two experiments are essentially parabolic in the entire dynamic range. In the recurrent model the ‘boosting’ of the salient stimuli thus appears to a large extent to be provided by recurrent excitation allowing for a partially linear activation function. In contrast, in the feedforward model the observed amplification of strong stimuli requires a purely parabolic activation function.

**Figure 8:**
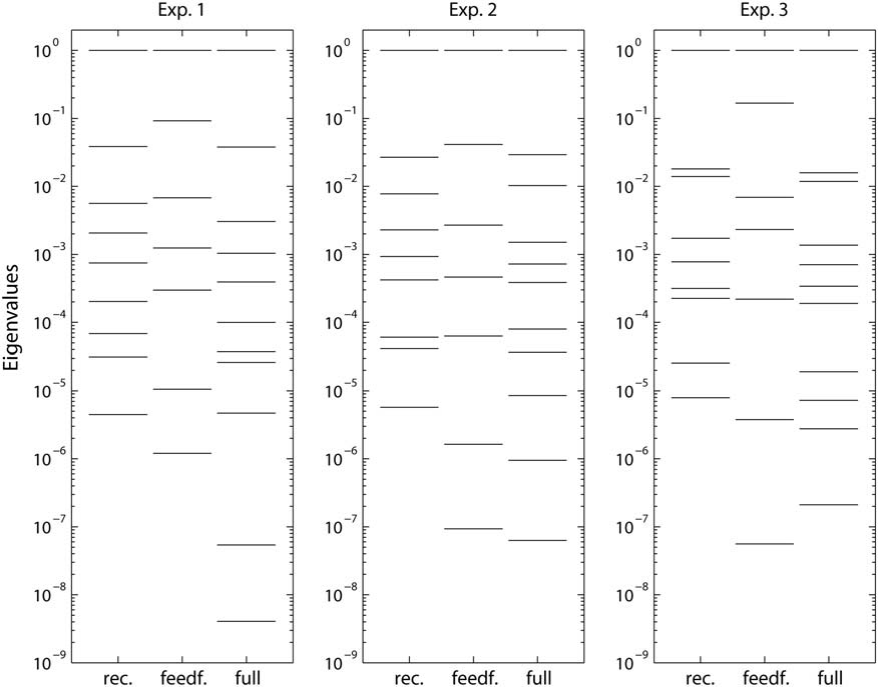
Sloppiness analysis of thalamocortical model. Illustration of parameter sensitivities, i.e., ‘sloppiness’, of model fits for recurrent (*rec.*), feedforward (*feedf.*) and full (*full*) thalamocortical models corresponding to Eqs. (2), (8), and (1), respectively. Eigenvalue spectra of the Levenberg-Marquardt Hessian 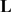 (Eq. 23) for experiments 1–3 are shown. The eigenvalues are normalized to the largest eigenvalue. Delay parameters 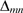 are left out of the analysis, since in our models these parameters are set to be discrete (with a time step of 0.5 ms).

Another way to compare the candidate thalamocortical models is to investigate their so called ‘sloppiness’ [27]. Sloppiness is a measure of the sensitivity of the model fit to changes in model parameters: large sloppiness means that some parameter combinations can be varied significantly without changing the quality of the model fit. Sloppiness can be quantified by an eigenvalue analysis of the Hessian matrix composed of the partial 2nd derivatives of the model error function [27],[29]. Here the so called Levenberg-Marquardt Hessian matrix 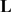 (Eq. 23) is used, cf. Materials and Methods. Fig. 8 shows the eigenvalue spectra of 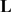 (23) for experiments 1–3 for the three investigated thalamocortical models: (1) the recurrent model (Eq. 2), (2) the feedforward model (Eq. 8), and (3) the full model including both feedforward inhibition and the recurrent connections (Eq. 1). The spectra are normalized so that the largest eigenvalue is unity. Small eigenvalues correspond to large sloppiness, and variations in model parameters along the direction of the corresponding eigenvectors in parameter space will have small effects on the overall model error [27]. The number of eigenvalues corresponds to the dimension of 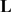. The feedforward delay parameters 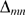, which are discrete parameters in the present modeling scheme, are absent in 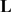 (cf. Materials and Methods), and the number of eigenvalues in Fig. 8 is thus nine for the recurrent model, seven for the feedforward model, and eleven for the full model.

In Fig. 8 we see more ‘sloppy’ eigenvalues for the feedforward model than the recurrent model. For example, only one of nine eigenvalues is found to be less than 2·10^−5^ (measured relative to the largest eigenvalues) for the recurrent model. In contrast, two of seven eigenvalues are less than 2·10^−5^ for the feedforward model. This larger ‘sloppiness’ is in accordance with the poorer fits observed for the feedforward model; the functional flexibility inherent in the feedforward model appears not to be well suited to account for the present data with 3 different amplitudes and 9 different stimulus rise times. For the full model we find three of eleven eigenvalues to be smaller than 10^−5^. Thus compared to the recurrent model, the added feedforward inhibition with two new parameters seems only to add two ‘sloppy’ eigenvalues, in line with the observation that the overall fit is not improved much.

To summarize, our results so far suggest that the model with recurrent inhibition accounts slightly better for the present experimental data than the model with feedforward inhibition, i.e., that the layer-4 interneurons providing the inhibition of the layer-4 excitatory neurons are in turn mainly driven by excitation from layer-4 neurons. Since the relative strength of recurrent effects compared to feedforward effects appears to increase with increasing thalamic stimuli [19], this might indicate that the present fits to the experimental data might be dominated by ‘strong’ inputs. To explore this further we investigated the properties of the thalamocortical transfer using linear-systems measures such as transfer functions in frequency space and impulse-response functions.

### Thalamocortical transfer

#### Thalamocortical frequency response

Our identified thalamocortical models are in general nonlinear due to the nonlinear activation functions 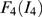. For small deviations around a working point, however, the above models can be linearized, so that linear-systems measures such as *transfer functions* in frequency space and *impulse-response functions* can be explored [30].

For the thalamocortical models the derivation of the transfer function amounts to deriving the response to a small sinusoidal modulation 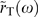 in the thalamic input superimposed on a constant background activity 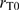. As shown in Materials and Methods the modulated response 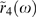 (complex notation) in layer 4 is then given by 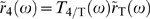 where 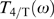 is the linear *transfer function* [30]–[32]. The transfer function for the full thalamocortical model Eq. (1) is derived in Materials and Methods, cf. Eq. (42), and for the *recurrent* model in Eq. (2) it reduces to
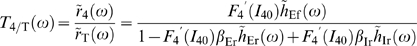
(9)


Here 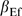 and 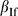 in Eq. (42) has been set to one and zero, respectively. 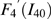 is the derivative of the activation function at the working point 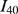, and the Fourier-transformed coupling kernels 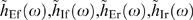 are all given by 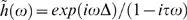, cf. Eq. (39).

This transfer function describes how a small modulation of the thalamic population firing rate transfers to the layer-4 population firing rate. The transfer function 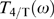 is a complex quantity where the transfer amplitude is given by the absolute value 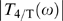, and the change in phase between the thalamic input and layer-4 output is given by the complex phase angle.

**Figure 9:**
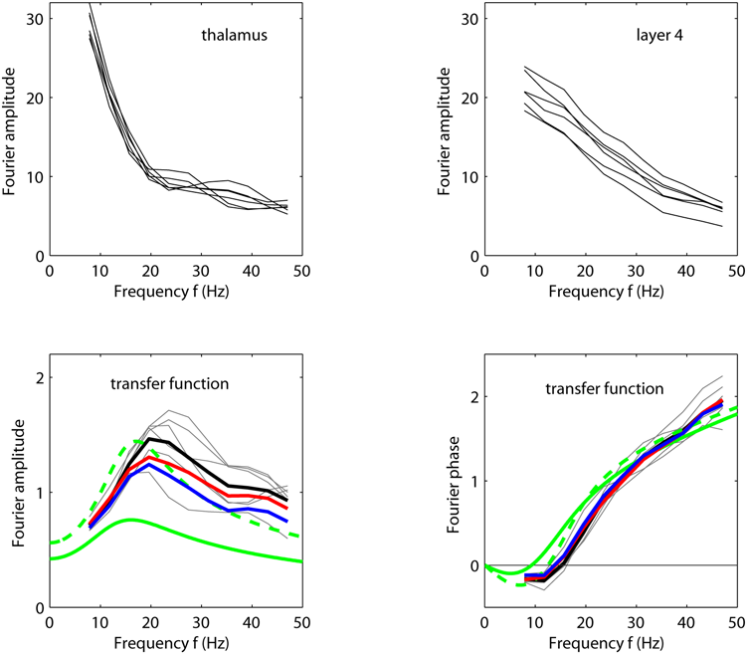
Thalamocortical transfer for experiment 1. Frequency content, i.e., Fourier amplitudes, of the thalamic input 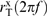 (upper left) and the layer-4 firing rate 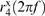 (upper right) for six stimulus conditions providing the strongest response in experiment 1 (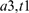; 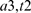; 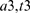; 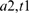; 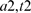; 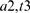). (Note that 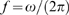.) The corresponding amplitude (lower left) and phase (lower right) of the experimental transfer ratio 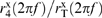 are shown as thin grey lines while the thick black lines represent the corresponding averages for these six stimuli. Examples of model predictions for amplitude (lower left) and phase (lower right) of the recurrent thalamocortical transfer-function model (Eq. 9) are shown for the fitted parameter values 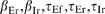 and 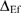 for experiment 1 taken from Table 1; green lines correspond to choosing 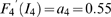, and dashed green lines to 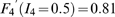. The red and blue lines in the lower panels correspond to the average of the amplitude (lower left) and phase (lower right) of the transfer ratios found for the six stimuli listed above when driving the fitted recurrent (red) and feedforward (blue) models in Eqs. (2) and (8), respectively, with the experimentally extracted thalamic input 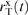.

Fig. 9 shows an example of how the Fourier amplitude (lower left) and phase (lower right) of the recurrent thalamocortical transfer-function model in Eq. (9) depends on frequency. The parameter values correspond to the fitted values of 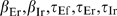 and 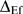 for experiment 1 taken from Table 1. Since the fitted activation function 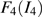 is nonlinear, there is no unique derivative 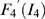 for the fitted model. In Fig. 9 we thus show results for two different values of the derivative: (i) 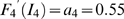 corresponding to the derivative on the linear flank, and (ii) 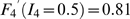 corresponding a larger derivative more characteristic for the parabolic part of the activation function. In both cases the amplitude of the model transfer function has its maximum at finite frequencies, 16 Hz and 18 Hz, respectively. This peak stems from a resonance-like phenomenon for frequencies where the denominator of Eq. (9) becomes small. Even larger values of 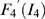 would increase the peak value further.

In Fig. 9 (lower right) we further observe that the *phase* of the transfer function is negative for the smallest frequencies. The negative transfer-function phase implies that the maximum in the layer-4 responses will precede the maximum of the thalamic input for a small sinusoidal input for these frequencies.

Fig. 9 further shows the amplitude of the Fourier components of the thalamic input 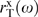 (upper left), the layer-4 firing rate 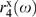 (upper right), as well as the amplitude (lower left) and phase (lower right) of the experimental transfer ratio 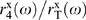 for six of the stimulus conditions providing the strongest response in experiment 1 (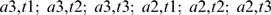). Also the average of the amplitudes and phases of the experimental transfer ratios across these six stimulus conditions are shown. The same characteristic resonance peak is seen in the experimental transfer ratios as for the linear-model transfer function in Eq. (9). The theoretical function for the largest shown value for 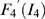, i.e., 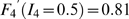, is seen to be closest to the experimental curves. This is not surprising since the depicted experimental curves correspond to six of the stimuli giving the largest responses for which also the parabolic part of the activation function is encountered. We see, however, that the maxima of the experimental transfer ratios are systematically shifted to somewhat higher frequencies compared to the predictions of the linear transfer-function model in Eq. (9). The same small deviation is also observed for the transfer ratio found by replacing 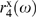 with the Fourier transform of the best fit 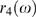 to the recurrent model in Eq. (2) (red curve in lower left panel of Fig. 9). Thus the deviation is presumably due to the non-linearities inherent in the system under the present stimulus condition, represented by the nonlinear activation function in the recurrent model. In Fig. 9 (lower right) we also observe a good qualitative agreement between the predicted phase difference between the thalamic input and layer-4 output from the linear transfer-function model and the experimentally measured phase differences.

The experimental stimulus set with 3 different amplitudes and 9 different stimulus rise times was found to provide the largest variation in neuronal responses and thus provide the best test data for distinguishing the two candidate models. As seen in Tables 1 and 2 the recurrent thalamocortical model give better fits for these experiments, i.e., experiments 1–3, than the feedforward model. The increases in the error measure 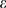 (Eq. 22) for the feedforward model compared to the recurrent model are between 20% and 60%. The results for the thalamocortical transfer in experiment 1 shown in Fig. 9 indicate that the apparent superiority of the recurrent models mainly lies in its ability to account for the rapidly varying parts of the measured layer-4 population response. As seen in the lower left panel of this figure, the ratio of the frequency contents of the experimentally extracted layer-4 and thalamic signals (solid line) is better accounted for by the recurrent model (red line) than the feedforward model (blue line) for frequencies above 20 Hz. For frequencies below 20 Hz the difference is less. For experiment 2, where the increase in error from the recurrent to the feedforward model is even larger, this tendency is even more pronounced (data not shown).

#### Thalamocortical impulse response

**Figure 10:**
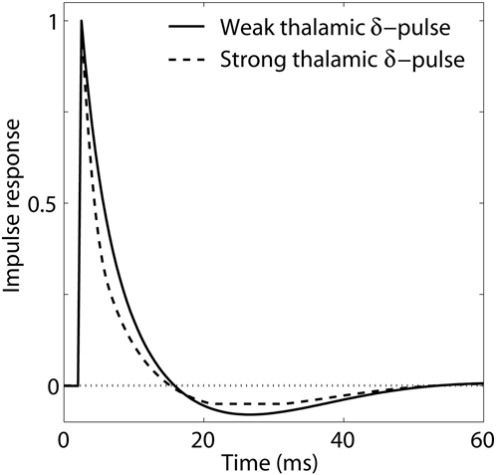
Thalamocortical impulse response for experiment 1. Impulse response for thalamocortical recurrent model (Eq. 2), i.e., additional response due to sharp ‘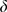-function’-like impulse, ‘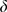-pulse’, of extra thalamic firing superimposed on the steady-state thalamic background activity. The parameter values from fitting to experiment 1 are used (cf. Table 1). The steady-state thalamic firing is set to corresponds to a steady-state layer-4 input ‘current’ 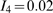, i.e., on the linear flank of the layer-4 activation function (cf. Fig. 4). Solid line corresponds to a linear impulse response, i.e., response to a sufficiently weak 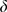-pulse so that only the linear part of the activation 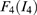 is encountered. Dashed line corresponds to a strong 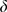-pulse of extra thalamic firing resulting in a maximum value of 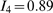, i.e., far into the non-linear region of the activation function.

Another traditional dynamical systems measure is the *impulse-response function*, i.e., the response to a very brief input signal [30]. Fig. 10 shows the impulse response of the recurrent thalamocortical model (Eq. 2) with model parameters fitted to experiment 1, cf. Table 1. In this context the impulse response is the additional evoked firing in the layer-4 population when adding a sharp impulse, ‘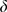-pulse’, to the background thalamic input. The solid line represents the impulse response for a background activity corresponding to the linear flank of the activation function 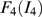 where the strength of the 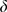-pulse is so small that the circuit remains in the linear regime. The observed impulse response is biphasic with an excitatory phase lasting about 15 ms followed by an inhibitory phase lasting up to 50 ms. The dashed line shows the response to a very strong 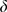-pulse so that the nonlinear regions of the activation function, both the parabolic and half-wave rectifying parts, are encountered. The response to this strong excitation is seen to be qualitatively similar to the linear impulse response. However, the excitatory phase is somewhat sharper, and the long inhibitory phase is modified by the half-wave rectification imposed by the requirement of nonnegative firing rates.

The biphasic thalamocortical impulse-response observed in Fig. 10 qualitatively resembles the temporal shape (i.e., excitation followed by suppression) of the ‘impulse-response’ to single whisker flicks observed in about two thirds of the barrel-cortex neurons in a single-unit study of Webber and Stanley [33]. The remaining third of the neurons in this study was found to have a second excitatory phase following the suppression [33]. It should be noted, however, that this ‘stimulus-cortical’ impulse-response is different from the thalamocortical impulse-response extracted here, since the ‘stimulus-cortical’ response also incorporates the processing between the whisker and the thalamus. A second excitatory phase in the ‘stimulus-cortical’ impulse response could thus be compatible with the observed biphasic thalamocortical impulse-response if the thalamic response to a single whisker flick has two temporally distinct positive phases (f.ex., due to cortical feedback onto the thalamic neurons).

### Intracortical model

Layer 4 is generally thought of as the dominant input layer for sensory activation of cortex. The supragranular population of layer-2/3 pyramidal neurons appears to receive a dominant input from the layer-4 population, and the initial stimulus-evoked response in layer 2/3 appears to largely stem from layer 4 neurons which in turn are activated by the thalamic input [11], [34]–[41]. We thus investigated models that could account for the experimentally extracted population firing-rate of the layer-2/3 population with the extracted layer-4 population rate as input. We initially described this activity using a combined feedforward and recurrent model analog to Eq. (1), i.e.,
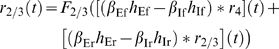
(10)


This form assumes that the interlaminar connections from layer 4 to layer 2/3 are predominantly provided by the excitatory neurons [38]. Fitting of this form to the experimental data revealed that the extracted population firing rates can be explained with a strongly reduced version incorporating only the excitatory feedforward term in Eq. (10), i.e.,
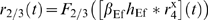
(11)


**Figure 11:**
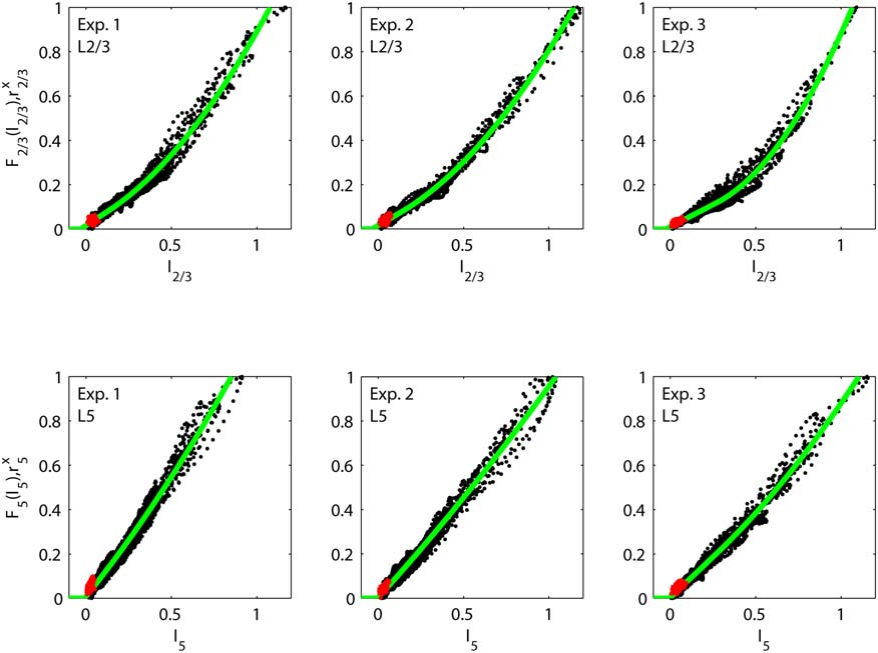
Fits of intracortical model. Illustration of fits of the intracortical model (Eq. 11) to data from experiments 1–3. Each dot corresponds to the experimentally measured layer-2/3 firing rate 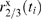 (upper row) or layer-5 firing rate 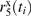 (lower row) at specific time points 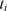 plotted against the fitted model values of 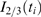 or 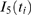, respectively. The red dots are the corresponding experimental data points taken from the first 5 ms after stimulus onset (for all 27 stimuli). These data points show the activity prior to any stimulus-evoked thalamic or cortical firing and represent background activity. The solid green curves correspond to the fitted model activation functions 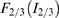 (upper row) and 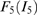 (lower row), respectively.

As before the weight 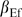 was set to unity without loss of generality. Further, the feedforward delay 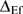 was found to be very small and consequently set to zero. Table 3 and Fig. 11 (upper row) show the results of fitting this model to the experimentally extracted layer-2/3 firing rates 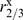 using the layer-4 firing rate 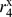 as input for experiments 1–3. A first observation is that the simple model in Eq. (11) accounts excellently for the experimental data; the error is less than 0.03 for all experiments. The fitted time constants 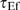 are all very short, less than 2 ms. The fitted activation functions for experiments 1 and 2 have significant non-linear, i.e., parabolic, contributions with shorter linear flanks than the corresponding activation functions for the recurrent thalamocortical model.

**Table 3:** Fitted model parameters for intracortical models.

L4→L2/3	Exp. 1	Exp. 2	Exp. 3
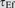 (ms)	1.2	1.2	1.8
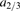	0.51±0.01	0.45	0.40
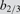	0.49±0.01	0.45	0.89
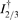	−0.03	−0.03	−0.02
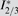	0.13±0.02	0.13	0.27
error ( 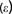 )	0.0214	0.0141	0.0279

Resulting optimized parameters for the intracortical network model (Eq. 11) for experiments 1–3. Upper row: Layer-2/3 firing rate predicted from layer-4 input. Lower row: Layer-5 firing rate predicted from layer-4 input (where the subscripts ‘2/3’ are replaced by ‘5’ in Eq. (11)). The listed parameter values correspond to the mean of estimated parameters from 25 selected models giving essentially the same error (see Materials and Methods). The standard deviations are only listed if they exceed the last digit of the mean.

A strong feedforward connection between the layer-4 and layer-2/3 populations is in accordance with previous experimental studies [34]–[36], and also with the results from the joint modeling of the present MUA data and the corresponding LFP data using *laminar population analysis (LPA)* in Einevoll et al. [11]. However, this LPA analysis also indicated a recurrent interaction within layer 2/3, in accordance with experimental observations by Feldmeyer et al. [42] of a substantial connectivity between layer-2/3 pyramidal neurons. Such a recurrent connection between excitatory neurons could amplify synchronous feedforward excitation from layer 4 [42]. In the present modeling study we find that no such excitatory recurrent terms are needed to account for the present stimulus-evoked experimental data. Instead the stronger inputs are amplified in our model by means of the boosting nonlinearity inherent in the activation function 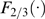, cf. Fig. 11. We therefore investigated to what extent recurrent connections, i.e., a model of the recurrent form in Eq. (10) with 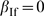, improved the fit for the situation with a *linear* activation function. In our test runs, it did not. For experiment 1 with linear activation function the error was found to only be reduced from 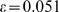 to 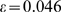 when adding recurrent excitation and inhibition. In comparison, the error for the simple feedforward model with a non-linear activation function was found to be only 0.021, cf. Table 3.

We also investigated to what the extent the extracted layer-5 population firing rate can be accounted for by the same type of firing-rate model with the layer-4 population providing the stimulus-evoked input [43],[44]. Table 3 and Fig. 11 (lower row) show the results of fitting a model of the type in Eq. (11) to the experimentally extracted layer-5 firing rates 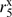 using the layer-4 firing rate 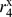 as input for experiments 1–3. Again we observed that the simple feedforward model accounts excellently for the experimental data with an error of less than 0.02 for all experiments. The fitted time constants 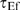 are again short, less than 3 ms. The most striking difference from the fits of the layer-5 firing rate is the almost linear activation functions. Thus the layer-5 firing rate appears to be related to the layer-4 firing rate in a very simple manner for the present experimental situation.

The presently extracted intracortical models have very simple mathematical forms. For example, for the layer-4 to layer-2/3 transfer the model on integral form can be translated to a simple differential equation using the ‘linear-chain trick’ as described in Materials and Methods, i.e.,
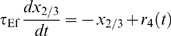
(12)where the layer-2/3 firing rate is given by 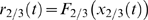. The same type of differential equation is found for the connection between layer 4 and layer 5 (with 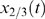 substituted by 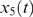 in Eq. (12)). In this case the signal transfer is essentially described by a simple linear differential equations for 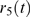 since 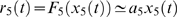.

### Intracortical transfer

We next investigated the intracortical transfer functions. The linear transfer function from the layer-4 population to the layer-2/3 and layer-5 populations is found from Eq. (11) to be given by
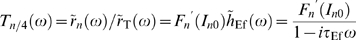
(13)where 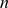 represents 2/3 or 5, and 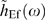 is found in Eq. (39). This corresponds to a low-pass filter, i.e., no resonance behavior as for the recurrent thalamocortical model in Eq. (9). Further, the impulse-response function is the simple (monophasic) exponentially decaying function, contrasting the biphasic thalamocortical impulse-response functions seen in Fig. 10.

**Figure 12:**
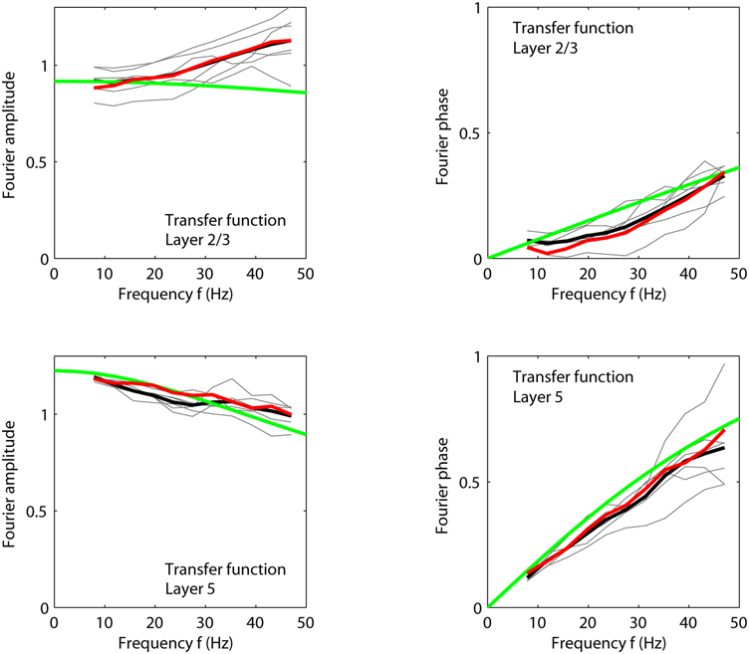
Intracortical transfer for experiment 1. Transfer ratios 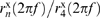 for six stimulus conditions providing the strongest response (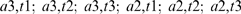) are shown as thin grey lines. (Note that 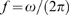.) Thick black lines represent the corresponding averages for the six stimuli. Upper left: Transfer to layer 2/3, amplitude. Upper right: Transfer to layer 2/3, phase. Lower left: Transfer to layer 5, amplitude. Lower right: Transfer to layer 5, phase. Examples of model predictions from the linearized intracortical transfer-function model in Eq. (13) are shown (dashed lines) for the parameter values corresponding to the fitted values of 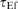 and 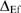 for experiment 1 taken from Table 3. The work-point derivatives were chosen to be 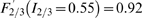 and 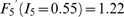, respectively. The red lines correspond to the average of the amplitude (left panels) and phases (right panels) of the transfer ratios found for the six stimuli listed above when driving the fitted feedforward intracortical layer-2/3 and layer-5 models of the type in Eq. (11) with the experimentally extracted layer-4 input 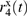.

In Fig. 12 we compare predictions for the transfer ratio from this simple linear feedforward model using the fitted parameters from Table 3 with the corresponding experimental result 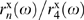 (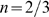 or 5) for six of the stimulus conditions providing the strongest cortical responses in experiment 1 (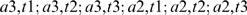). For the layer-5/layer-4 transfer ratio (lower row in Fig. 12) we see an excellent agreement between experiments and linear theory. This is as expected since the fitted layer-5 activity functions 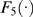 are essentially linear (cf. Fig. 11). For the transfer ratio between layer 2/3 and layer 4 a more substantial deviation between the experimental and linear-model transfer rations is observed, in particular a slightly growing transfer-ratio amplitude for large frequencies. This deviation presumably reflects the non-linearities of the layer-2/3 activity functions 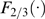, because the same effect is also observed for the transfer ratio between the best model fit 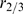 and 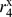 (cf. red line in upper left panel of Fig. 12).

## Discussion

A main objective of the present paper is the description and application of a new method for extraction of population firing-rate models for thalamocortical and intracortical signal transfer on the basis of trial-averaged data from simultaneous cortical recordings with laminar multielectrodes in a rat barrel column and single-electrode recordings in the homologous thalamic barreloid. Below we first discuss the results from the model estimation for both the thalamocortical and the intracortical signal transfer, followed by a discussion of the model estimation method itself.

### Thalamocortical models

For the thalamocortical signal transfer our investigations clearly identifies a model with (1) fast feedforward excitation, (2) a slower predominantly inhibitory process mediated by recurrent (within layer 4) and/or feedforward interactions (from thalamus), and (3) a mixed linear-parabolic activation function. The relative importance of the feedforward compared to the recurrent connections is more difficult to determine and has been a subject of significant interest [7]–[9],[16],[45]. Here we have compared two alternative models: (i) a purely *feedforward* thalamocortical model with feedforward excitation and inhibition and (ii) a *recurrent* model with feedforward excitation, recurrent excitation and recurrent inhibition (but no feedforward inhibition). In reality the inhibitory layer-4 neurons receive excitatory inputs both from thalamic neurons and other layer-4 neurons, and the inhibition felt by the excitatory layer-4 neurons will thus be both ‘feedforward’ and ‘recurrent’; the different models thus explore what part appears to dominate in the present experimental situation.

Our results suggest that the model with recurrent inhibition better accounts for the present experimental data than the model with feedforward inhibition, i.e., that the layer-4 interneurons providing the inhibition of the layer-4 excitatory neurons are in turn mainly driven by excitation from layer-4 neurons. Since the relative strength of recurrent effects compared to feedforward effects appears to increase with increasing thalamic stimuli [19], this might indicate that the present fits to the experimental data is dominated by ‘strong’ inputs. Alternatively, it may be that a strong feedforward inhibition is also present, but that it is strongly overlapping in time with the feedforward excitation. Then the present modeling scheme and experimental data are unable to separate them and instead lump the effect of this inhibition together with the feedforward excitation.

An interesting result from the present investigation is that we find a similar thalamocortical population model as Pinto et al. [8],[9]: the *recurrent* model on integral form in Eq. (2) can be mapped to a set of two differential equations (Eqs. 4–5) structurally similar to the model suggested and investigated by Pinto and coworkers. The correspondence between the behavior of these models is demonstrated by the phase-plane plots in Fig. 5, which exhibits the same qualitative features as the analogous phase-plane plots in Ref. [9]. This close correspondence is striking since the methods used to derive the models are very different. Pinto et al. chose the model to account for a large collection of single-unit PSTHs recorded with sharp electrodes from numerous animals. By contrast, our model parameters were extracted based on simultaneous thalamic and cortical recordings from individual animals. As multielectrode arrays allow for convenient simultaneous recordings from all layers of the cerebral cortex, even at more than one cortical location [10], the proposed modeling approach scales more readily to complex networks involving multiple, spatially separated, neuronal populations.

### Intracortical model

The presently identified intracortical model for the connection between the layer-4 and layer-2/3 populations has a very simple mathematical form including only a single feedforward term (Eqs. 11–12), but was nevertheless found to account excellently for the experimental data, cf. Fig. 11. Such a strong feedforward connection between these populations is in accordance with previous experimental studies [34]–[36]. An experimental study by Feldmeyer et al. [42] found substantial recurrent connectivity between layer-2/3 pyramidal neurons, but no such excitatory recurrent terms were needed to account for the present stimulus-evoked experimental data. Compared to the thalamocortical transfer, the intracortical transfer displayed limited variation for the present set of stimuli under the present experimental conditions. The absence of identified recurrent terms in our intracortical models may thus be due to lack of sufficient variance in the experimental data to allow for identification of such a model term. Instead recurrent intracolumnar interactions in layer-2/3 may be involved in shaping the non-linear activation function 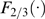 and thus modifying the dynamics in a way which cannot be accounted for by the present recurrent model with a linear activation function.

As seen in Fig. 11 and Table 3 a simple feedforward model can, based on the layer-4 population firing rate, also account excellently for the observed layer-5 population firing rate. As for the layer-4 to layer-2/3 pathway, the fitted time constants are quite short, 3 ms or less. Interestingly, the fitted activation function is found to be almost linear (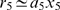, cf. (12)), so that the dynamics of the layer-5 population firing rate can be described simply by 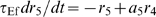. Monosynaptic connections between pairs of spiny stellate cells in layer 4 onto the basal dendrites of layer-5 pyramidal cells compatible with a such a fast feedforward connection have been observed experimentally [43],[44]. Moreover, results from the LPA analysis of the present data in Einevoll et al. [11] were compatible with such an excitatory synaptic connection from layer 4 onto the basal layer-5 neuron dendrites. However, the presently extracted intracortical firing-rate model may simply reflect that extracted firing rates for the cortical populations are strongly overlapping in time. An alternative explanation for the observed tight temporal correlation between the layer-4 and layer-5 firing rates could be correlations between the input from thalamic neurons in VPM onto layer 4 with VPM inputs to layer-5B neurons [46] and/or ‘paralemniscal’ input via POm to layer 5A neurons [39],[47]. Thus while the thalamocortical transfer seems to be well probed by the present stimulus paradigm, i.e., the set of stimuli provides a varied set of paired thalamic and layer-4 responses, this is less so for the intracortical transfer. One should thus search for other experimental paradigms to probe the intracortical population-rate dynamics further.

As a methods test we also investigated to what extent our feedforward model could account for the ‘backwards’ connections, i.e., whether the layer 4 firing could mathematically be accounted for by the model in Eq. (11) with the extracted layer-2/3 or layer-5 population firing rates as input respectively. These connections are expected to be weak [37],[41], and indeed, since the layer-4 firing initiates somewhat earlier than the rest of the laminar populations, the best fits were found for unrealistically small time constants (*τ*_Ef_<0.1 ms). The model solutions naturally failed to predict the onset of the layer-4 firing correctly, indicating their inadequacy.

### Coding properties of thalamocortical and intracortical circuits

A conclusion from our study is that the signal processing in the thalamocortical and intracortical circuits is very different. The thalamocortical circuit favors rapid whisker movements in that sharply rising thalamic population firing rates evokes large cortical responses. For such stimuli the fast excitation in the circuit dominates the slower, but eventually stronger, inhibition at short time scales thus ensuring a large population firing-rate in the layer-4 population for a certain time window. For slower whisker movements the inhibition overtakes the excitation before a strong cortical response has emerged. This essential feature is demonstrated by the phase-plane plots in Fig. 5. In frequency space the effect translates into a resonance-like amplification of the transfer function at intermediate frequencies (∼15–20 Hz), cf. Fig. 9, while the impulse response function is biphasic, cf. Fig. 10. In terms of coding the thalamocortical transfer can be said to map time derivatives of the thalamic population firing rate onto amplitudes in the population firing of cortical layer 4 [9],[16]. Thus the thalamocortical circuit is sensitive to angular whisker velocity, explaining why a rich variety of cortical responses are observed for the stimulus set with three different amplitudes and nine different rise times.

The intracortical circuits appear very different. Here the extracted models perform low-pass filtering, but the time constants are so short, typically 1–3 ms, that the cutoff frequency is large: for firing-rate frequencies up to 50 Hz only modest reductions in the transfer ratio is observed, cf. Fig. 12. In contrast to the typical biphasic form seen for the impulse response for the thalamocortical model in Fig. 10, the impulse response of the intracortical models is a rapid exponentially decaying (monophasic) function. Thus, while the thalamus to layer-4 circuit appears to selectively respond to synchronous inputs with large time derivatives, the layer-4 to layer-2/3 circuit appears to perform a simple amplitude mapping [16].

### Current limitations and potential future extensions

A core assumption in our modeling scheme is that the MUA signal is proportional to the population firing rate [48],[49]. This assumption was recently investigated by Pettersen et al. [12] in a forward-modeling study of extracellular potentials from a population of layer-5 pyramidal cells from cat visual cortex receiving synchronous synaptic activation resembling the present stimulus-evoked situation. For *trial-averaged* data a roughly linear relationship was observed for a large range of small and intermediate population firing rates. For large population firing rates the true population firing rate was found to grow superlinearly with the MUA due to increasing cancelation of extracellular potentials for high firing rates. It is, however, unclear whether or to what extent such a superlinear relationship can be expected here.

A number of factors in our study could affect the magnitude and frequency content of the recorded electrophysiological response in comparison with awake behaving animals in a natural environment. Among them are anesthesia conditions and the use of an artificial single-whisker stimulus in the absence of active whisking. The present study used alpha-chloralose, an anesthetic agent used widely in neurophysiological and hemodynamic studies due to its lesser effects on cardiovascular, respiratory and reflex functions [50]–[52]. Qualitatively similar laminar profiles of evoked MUA response was observed with ketamine [11], but a systematic study is needed to investigate the effects of anesthesia on the identified network models.

Presently we have assumed the measured MUA to correlate with the excitatory population. Due to their lower neuron numbers the inhibitory populations are expected to contribute less, although differences in morphology and firing rates between excitatory and inhibitory neurons will also affect their relative contributions [13]. More forward-modeling studies along the lines of Pettersen et al. [12] are needed to investigate this relationship between the measured MUA and the underlying neural activity in intermixed excitatory and inhibitory populations.

In the current models only the firing rates of the excitatory laminar populations are modeled explicitly, while the inhibitory neurons are merely considered to modify the dynamics of, and interaction between, these excitatory populations. An alternative to the single population Volterra firing-rate model for the thalamocortical transformation in Eq. (1) could be the following generalization including an explicit dynamical equation also for the inhibitory firing rate, i.e.,
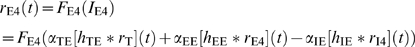
(14)

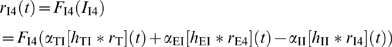
(15)


This model explicitly includes thalamic input to both the excitatory and inhibitory populations as well as all possible recurrent connections [9]. The two integral equations in Eqs. (14–15) can be mapped to a set of four differential equations using the ‘linear-chain trick’ [14]. However, as shown in Materials and Methods, the model in Eqs. (14–15) can be reduced to our model in Eq. (1) under the assumptions that (1) the inhibitory firing-rate function is linear, i.e., 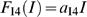, (2) the self inhibition is zero, 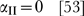 [53], and (3) by making the identifications 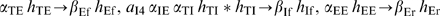, and 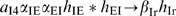, cf. Eqs. (43–47).

The analysis of model parameter identifiability revealed that certain linear combinations of parameters (notably recurrent and feedforward excitation and inhibition strengths, time constants, and activation function parameters), do not have a measurable effect on the measured signals, and thus cannot be confidently estimated. This problem can be partly addressed by using intracellular recordings to measure some of these parameters, e.g., synaptic time constants and firing rates as function of somatic input currents, directly in the relevant cell types. Model identification could also be aided by theoretical investigations of the basic structure of population firing-rate models [54],[55].

Furthermore, intercolumnar signal propagation can be modeled through connecting single-column firing-rate models of the present type based on known connectivity between neighboring columns or commissural projections [56]–[62]. Such multicolumn models may provide insights into the mechanisms of more complex cortico-cortical interactions such as surround [63],[64] and transcallosal inhibition in SI [65]–[67].

Several groups have attempted to derive information about functional connectivity between cortical areas based on non-invasive imaging data [68],[69]. A further development of this approach, aimed at providing a general theory of information processing in hierarchies of cortical areas, has recently been proposed by Friston [70],[71]. The interpretation of the results from such high-level modeling approaches has been limited by the lack of a biophysical measurement theory relating the imaging signals to the underlying neuronal population activity and the connections between neuronal populations. The modeling approach described here could provide the necessary biophysical grounding for such theories, as the noninvasive electroencephalography (EEG) and magnetoencephalography (MEG) signals can be directly predicted based on the laminar distribution of current sources and sinks, along with information about the geometry of the cortical surface of the individual subject [72],[73]. Thus, by embedding the proposed ‘mesoscopic’ modeling approach within a macroscopic modeling framework, such as the Hierarchical Dynamical Model (HDM) proposed by Friston [71], it may ultimately be possible to bridge the gap from the cellular to the systems-level of description, and from invasive to non-invasive recordings.

## Materials and Methods

### Experimental procedure

All experimental procedures were approved by the Massachusetts General Hospital Subcommittee on Research Animal Care. Male Sprague-Dawley rats (250–350 g, Taconic) were used in the experiments. Glycopyrrolate (0.5 mg/kg, i.m.) was administered 10 minutes before the initiation of anesthesia. Rats were anesthetized with 1.5% halothane in oxygen for surgery. Following surgery (see below), halothane was discontinued, and anesthesia was maintained with 50 mg/kg intravenous bolus of alpha-chloralose followed by continuous intravenous infusion at 40 mg/kg/h. During surgery a tracheotomy was performed, cannulas were inserted in the femoral artery and vein. All incisions were infiltrated with 2% lidocaine. Following tracheotomy, rats were mechanically ventilated with 30% O_2_ in air. Ventilation parameters were adjusted to maintain the following blood gas readings: PaCO_2_ between 35 and 45 mm Hg, PaO_2_ between 140 and 180 mm Hg, and pH between 7.35 and 7.45. Heart rate, blood pressure, and body temperature were monitored continuously. Body temperature was maintained at 37.0±0.5°C with a homeothermic blanket (Harvard Apparatus, Holliston, MA, USA). The animal was fixed in a stereotaxic frame. An area of skull overlying the primary somatosensory cortex was exposed and then thinned with a dental burr. The thinned skull was removed and the dura matter dissected to expose the cortical surface. A barrier of dental acrylic was built around the border of the exposure and filled with saline.

Mapping with a single metal microelectrode (FHC, 2–5 MΩ) was done to determine the positioning of the linear (laminar) multielectrode array in barrel cortex. The optimal position was identified by listening to an audio monitor while stimulating different whiskers. Following the mapping procedure, the electrode was withdrawn, and the laminar electrode was slowly introduced at the same location perpendicular to the cortical surface. Contact no. 1 was positioned at the cortical surface using visual control with saline covering the exposed cortex around the laminar electrode. The linear multielectrode had 23 contacts with diameter 0.04 mm spaced at 0.1 mm [49], and data from contacts 2 to 23 was used in the further analysis.

The thalamic recordings were performed using single metal microelectrodes (FHC, 5–7 MΩ). The ventral posteriomedial thalamic nucleus (VPM) was targeted using stereotactic coordinates (VPM: AP −3.6 to −3.0, ML 2.0 to 3.5, DV 5.0 to 7.0). VPM (rather than POm) was chosen because it provides the dominant input to cortical layer 4 [74].

Single whiskers were deflected upward by a wire loop coupled to a computer-controlled piezoelectric stimulator. The stimulus sequence was optimized using the approach described by Dale [75]. This method optimizes efficiency for Finite Impulse Response (FIR) estimation, and, thus, makes no a priori assumption of the response shape. Stimuli were presented at a fixed rate of one per second, with 25% of stimuli being ‘null events’ (zero amplitude whisker flicks). The method was used to optimize the order of stimulus conditions so as to optimize estimation efficiency of the event-related neuronal responses. We used two stimulus conditions: In the first stimulus condition, with altogether 27 different stimuli, three stimulus amplitudes (vertical displacements 0.40 mm (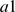), 0.80 mm (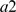), and 1.2 mm (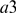)) each with nine stimulus rise times (20 ms (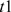), 30 ms (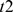),…, 100 ms (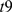)) were applied. The stimulus angular velocity varied between 76 deg/s (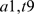) and 1090 deg/s (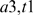). In the second stimulus condition a fixed stimulus rise time (time from onset to maximum displacement) of 30 ms was used, with 27 different, linearly incrementing, vertical displacements up to 1.2 mm (amplitude 27).

### Preprocessing of experimental data

The recorded laminar-electrode potential was amplified and analogically filtered online into two signals: a low-frequency part and a high-frequency part [49]. Only the high-frequency part (150–5000 Hz, sampled at 20 kHz with 12 bits) was used in the present analysis. This signal was further filtered digitally between 750 Hz and 5000 Hz using a zero phase-shift second-order Butterworth filter, and then *rectified* along the time axis to provide the multi-unit activity (MUA). This non-negative high-frequency ‘envelope’ reflects firing of action potentials [12],[48],[49]. The time-resolution of this MUA signal was then decimated by a factor 10, reducing the time resolution of the data from 0.05 ms to 0.5 ms [11].

Sample traces of the laminar-electrode MUA (prior to rectification) for a single trial can be seen in Figure 1B in Einevoll et al. [11]. The process of (1) band-pass filtering (750–5000 Hz), (2) rectification, and (3) decimation was also applied to the thalamic single-electrode recordings to provide the thalamic MUA signal. The presently used *trial-averaged* data were obtained by averaging over all 40 trials for each stimulus type.

Six experiments recorded from a total of three rats were considered. In experiments 1–3 the stimulus condition with 3 amplitudes and 9 rise-times was used, while the 27-amplitude stimulus condition was used in experiments 4–6. Experiments 1, 2, 4 and 5 were from a single rat, while experiments 3 and 6 were from two other rats. About ten (out of 6480) trials were removed due to artifacts in the laminar-electrode MUA. In experiments 3 and 6, the laminar-electrode MUA signal from contact no. 9 was erratic; for this channel the arithmetic mean of the MUA signals from the neighboring contacts (contacts no. 8 and 10) was therefore used instead. The trial-averaged MUA data used for the estimation of cortical population firing rates is denoted 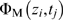. 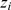 and 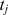 refer to the electrode position and time, respectively, and the indices 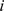 and 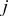 run over electrode contacts and time points.

### Estimation of thalamic population firing rate

The MUA recorded by the single thalamic electrode was used as a measure of the population firing rate of thalamic neurons in a barreloid in VPM projecting onto the homologous barrel. An estimate of the trial-averaged thalamic firing rate 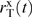 was found by an additional low-pass filtering of the MUA using a zero phase-shift, third order Butterworth filter with a cutoff frequency of 200 Hz. With this filter a 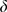-function is transformed to a peak with a half-width of about 2 ms. The value of the resulting rate signal was then shifted and normalized in each experimental data set separately so that the minimum and maximum estimated (normalized) thalamic firing rate over all times and stimulus conditions was zero and unity, respectively.

### Estimation of cortical population firing rates

The estimation of population firing rates of laminar cortical populations from laminar-electrode MUA is less straightforward. However, Einevoll et al. [11] recently developed a new method, *laminar population analysis (LPA)*, for analysis of laminar-electrode data. Using this method, laminarly organized neuronal populations can be identified and the time dependence of the population firing rates estimated. The laminar-electrode MUA data 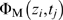 are then modeled as a sum over spatiotemporally separable contributions from several neuronal populations, i.e.,
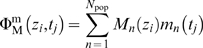
(16)where 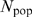 is the number of populations, 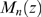 is the MUA spatial profile related to firing of action potentials in neuronal population 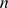, and 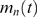 relates directly to the corresponding time course of firing activity in this population. With additional physiological constraints imposed on the general shape of 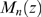, both 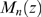 and 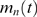 can, as described in Einevoll et al. [11], be determined in an optimization scheme minimizing the square deviation between 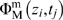 and the experimental data 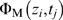. In Einevoll et al. [11] four distinct cortical populations were identified and putatively interpreted as layer 2/3, layer 4, layer 5, and layer 6 (and/or deep layer 5) populations. Experiments 1, 3, 5, and 6 correspond to data also analyzed there, and fitted results for 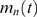 from this study were applied directly. For illustration of the correspondingly estimated spatial profiles 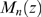, see Fig. 12 in Ref. [11]. For experiments 2 and 4 new LPA analyses were carried out to extract 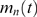.

**Figure 13:**
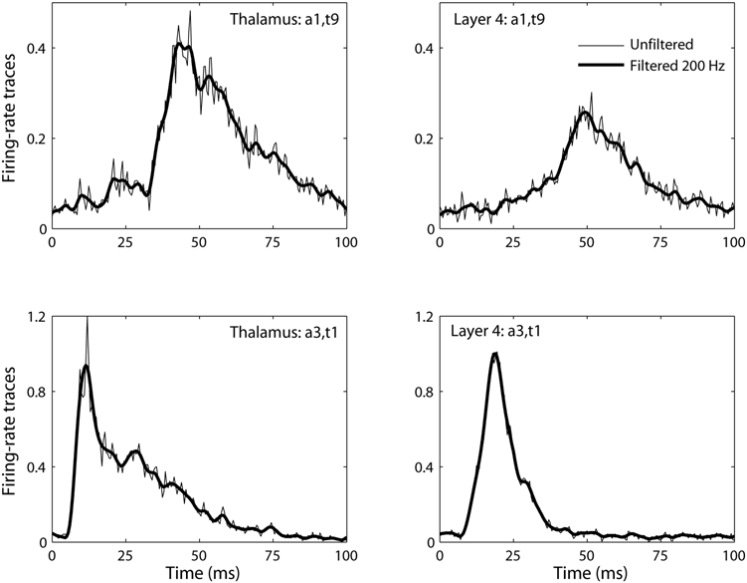
Effects of filtering on population firing-rate estimates. Example trial-averaged firing-rate traces, both unfiltered and filtered, from experiment 1 for the thalamic and layer-4 populations for a weak (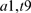) and a strong stimulus (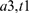). The unfiltered layer-4 traces correspond to 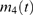, cf. Eq. (16), while the layer-4 filtered traces correspond to the firing-rate estimate 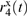 described in Eq. (17). The unfiltered thalamic traces represent the raw single-electrode MUA data. The relationship to the thalamic firing-rate estimate 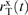 is described by Eq. (17).

Pettersen et al. [12] recently investigated the relationship between action-potential firing in a neuronal population and the MUA recorded by adjacent laminar electrodes. With a forward modeling scheme they calculated the extracellular potential generated by a columnar population of layer-5 pyramidal cells following synaptic activation resembling the stimulus-evoked situation investigated here. They found that a filtered version of the raw MUA in general gave a good measure of the population firing rate, known exactly in their model situation, for the trial-averaged case. Their study thus support the use of Eq. (16). Further, in their forward-model study a Gaussian filter with a standard deviation of 1 ms was found to be suitable. Presently, the signal was low-pass filtered at 200 Hz with a zero phase-shift, third order Butterworth filter, giving a similar temporal filter width. Fig. 13 demonstrates the effect of this low-pass filtering on example raw MUA thalamic and layer-4 population signals both for a weak (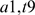) and a strong (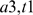) stimulus condition.

The resulting filtered estimates
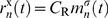
(17)were then shifted and the constant 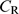 adjusted so that in each experimental data set the minimum firing rate was zero and the maximum firing rate unity for each identified cortical population. Here the superscript ‘
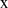
’ denotes that the firing-rate estimates stem from experiment, and the superscript ‘
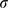
’ denotes that the signal has been low-pass filtered.

We assume that 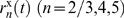 is a measure of the firing rate of laminar populations of excitatory neurons. For example, 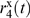 is interpreted as the population firing rate of the excitatory neurons in layer 4 of the barrel column. There will certainly also be contributions to the MUA from the inhibitory neurons, but since more than 80% or neurons in barrel cortex are excitatory [39] and the excitatory and inhibitory neurons in layer 4 have been observed to have firing probabilities with similar size and temporal profile when stimulated by the principal whisker [18], we assume the recorded signal to correlate well with the firing-rate of the excitatory population.

### Form of population firing-rate model on integral form

The population firing-rate models are formulated as nonlinear Volterra integral equations [5],[14],
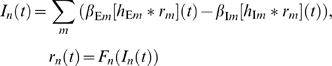
(18)where 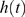 is a temporal coupling kernel, and 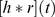 is the temporal convolution given by
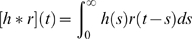
(19)


The quantity 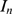 can be interpreted as net input current entering the somas of neurons belonging to population 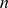. This current is assumed to result from previous firing in all presynaptic populations 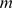 (possibly including 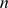 itself), with 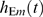 and 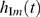 describing the excitatory and inhibitory effects, respectively, of firing at different times in the past. Presently, the temporal coupling kernels 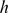 are modeled as (normalized) delayed decaying exponentials, i.e.,
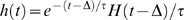
(20)where 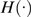 is the Heaviside unit step function. This kernel is specified by two parameters, the time constant 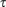 and the time delay 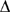.

In our model the net input current 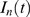 is converted instantaneously into a population firing rate 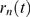 by means of a nonlinear function 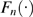. Here the following four-parameter class of activation functions is used:
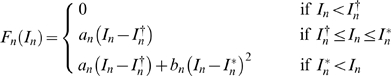
(21)


As illustrated in Fig. 3A this activation function is zero below the threshold 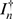, grows linearly between 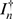 and 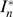, and quadratically above 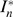. The activation function is continuous, and the positive coefficients 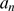 and 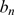 determine the steepness of the linear and quadratic term, respectively. Note that for 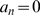 (or 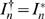) the linear part of the activation vanishes. Likewise, for 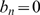 (or 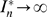) the activation above threshold is linear.

### Numerical optimization procedure

The model parameters were estimated by fitting model estimates of the population firing rates 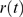 to the corresponding experimentally extracted rates 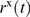 for all stimulus conditions simultaneously. The squared deviation between model and experimental firing rates was used as error measure, i.e.,
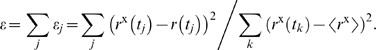
(22)


Here the sums over 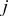 and 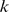 go over all time steps, i.e., 201 for each stimulus multiplied by the 27 different stimuli, and 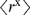 denotes the temporal mean of 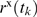.

The numerical calculations were done using MATLAB. In the numerical evaluation of the thalamocortical model (Eq. 1) the experimentally extracted thalamic firing rate 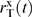 was inserted directly. The convolutions in the feedforward terms on the right hand side of Eq. (1) could thus be performed once and then stored for use in the later numerical procedure. For the recurrent term this is not possible since 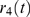 is evaluated iteratively for each time step. The firing rate 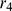 at time 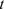 is given by the right hand side of Eq. (1) with the recurrent convolution terms evaluated at time 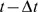 where Δ*t* = 0.5 ms is the time step. As a result, the recurrent synaptic connections can be considered to have a fixed time delay of 0.5 ms. The convolutions with the exponential coupling kernels were approximated using MATLAB's FILTER command. No explicit delays were considered for the recurrent terms, but for the feedforward terms delays corresponding to multiples of the time step (0.5 ms) were allowed for.

A three-step optimization procedure was used. In the first step the full model was considered and a preliminary optimization was performed doing a stochastic search starting from several sets of random initial parameter values 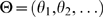 where 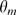 represent individual model parameters. We then computed linear correlations between the parameters for an ensemble of fitted models 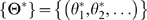 providing best fits, i.e., lowest values of the error 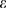 in Eq. (22), from this initial search. These correlations were then used to temporarily reduce the number of independent parameters to be used in a second stage of the optimization. For this second stage initial parameter values were chosen randomly from a distribution around the mean parameter values obtained from the ensemble of first-stage fits. After the second round of optimization in the reduced parameter space, all model parameters were again allowed to vary independently in the full parameter space. In this third part of the optimization procedure the initial values were taken to be the parameters resulting from the second-stage run. MATLAB's FMINSEARCH command, based on the Nelder-Mead algorithm, was used at the second and third stages of the optimization procedure. After the establishment of the ensemble of first-stage fits, a set of 25 independent second (and subsequent third) stage optimizations was run. The optimization procedure terminated if all 25 optimization jobs obtained the same minimum error value within 0.0001. If not, the procedure starting from the second stage, was repeated.

### Analysis of model ‘sloppiness’

In the optimization procedure we take advantage of linear correlations in the ensembles of fitted model parameters. Principal component analysis (PCA) of the covariance matrices 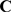, where 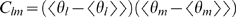 and 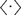 denotes the ensemble average, showed a substantial interdependency between these fitted parameters. More than 99% of the variability of the fitted parameters was typically accounted for by the first two components. As a consequence, large variations in the sets of optimal parameters may leave the overall quality of the fit, i.e., the error measure, almost unaffected as long as they are restricted to the subspace spanned by the dominant principal components. This property is called *sloppiness*, and can be quantified by an eigenvalue analysis of the Hessian matrix composed of the partial 2nd derivatives of the model error function evaluated for the estimated optimal (minimum error) parameter combination [27],[29]. To evaluate the sloppiness of our various candidate models the so called Levenberg-Marquardt approximation 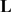 of the Hessian, defined via [27],[29]

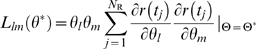
(23)was used, where the derivatives were evaluated for the overall best parameter set 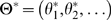. Here 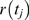 is the model firing rate at time 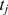, and the sum goes over all 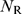 data points, in our case 201×27 = 5427. Note that the delay parameters 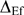 (and 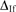 for the models with feedforward inhibition) were left out of the analysis, since in our models the smallest possible step in this parameter was 0.5 ms. The depicted eigenvalues of 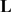 shown in Fig. 8 were normalized by the maximum eigenvalue.

The spectrum of these eigenvalues can be related to the shape of an ellipsoidal constant-error surface in a high-dimensional parameter space. The length of each principal axis of this constant-error surface is inversely proportional to the square root of the corresponding eigenvalue [27]. Directions with large eigenvalues are thus ‘stiff’ in the sense that the error increases rapidly in this direction. In contrast, in the ‘sloppy’ directions, corresponding to small eigenvalues, the error value changes little.

### Thalamocortical model as differential equations

#### Derivation of differential equations

The full thalamocortical model on integral form in Eq. (1) can be mapped to a set of two differential equations by means of the ‘linear chain trick’ [5],[14]. This model can be written as
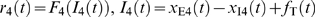
(24)where the auxiliary variables 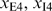 and 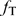 are given by
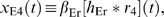
(25)

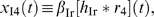
(26)

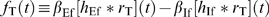
(27)


Since the temporal coupling kernels all are given as exponentially decaying functions, differentiation of the expression for 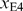 in Eq. (25) and 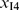 in Eq. (26) with respect to time yields [14],[76]

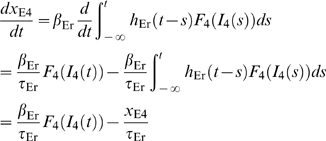
(28)

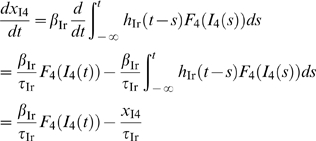
(29)


Here we have used that the delay constants 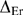 and 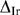 in the recurrent coupling kernels 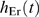 and 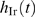 are set to zero.

#### Stability analysis of thalamocortical model

On differential form the stability of the thalamocortical model can be directly assessed. For the situation with a stationary input 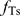, the differential equation system in Eqs. (28–29) is given by
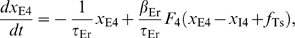
(30)

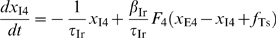
(31)


The Jacobian of this autonomous equation system is given by
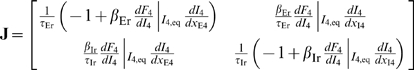
(32)where 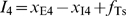. To ensure stability of the equilibrium point for this two-dimensional system, a negative trace tr(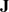) and a positive determinant det(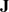) of the Jacobian is required (see, e.g., Wyller et al. [28]). For our activation function in Eq. (21) we have
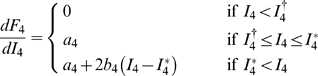
(33)


For the situation where the equilibrium point corresponds to 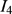 on the linear flank of the activation function, we thus have 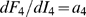 at the equilibrium value 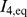. We then find
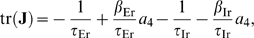
(34)

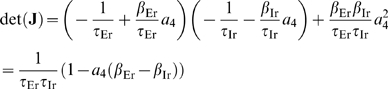
(35)where we have used that 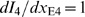 and 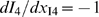. The requirements of a negative-trace and a positive-determinant for stability of the equilibrium point thus translate to the inequalities in Eqs. (6–7) in the main text.

These expressions can be directly generalized to the case where the background equilibrium state is on the parabolic part of the activation function by replacing 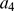 by 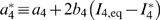. Without recurrent excitation, i.e., 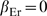, stability is always assured.

### Thalamocortical transfer function

Our thalamocortical models are in general nonlinear due to the nonlinear activation function 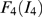 described by Eq. (21). However, a linear transfer function can be found by linearization around a working point, i.e., by deriving the response to a small sinusoidal modulation 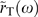 in the thalamic input population firing rate superimposed on a constant background firing rate 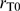. Following a standard perturbative approach we make the ansatz
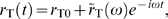
(36)

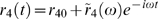
(37)


With these expressions the convolutions in Eq. (1) can be rewritten as
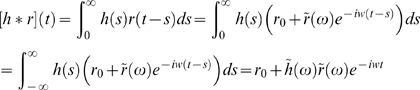
(38)where we have used that (i) 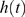 is normalized, (ii) 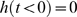 by construction so that the lower boundary in the integral above can be set to 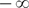, and (iii) introduced the Fourier transform 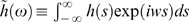. For our exponentially decaying coupling kernels in Eq. (20) we further find
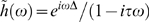
(39)


We now assume the perturbations in the input 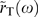 to be small and make the Taylor expansion
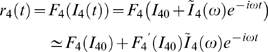
(40)so that we can identify 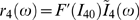. By using the general mathematical result in Eq. (38) in the model expression Eq. (1) we thus find
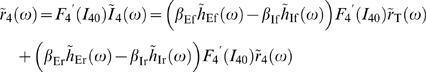
(41)


Algebraic rearrangement of this expression gives 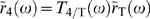 where 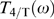 is given by
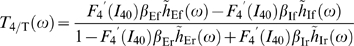
(42)


### Reduction of two-population thalamocortical model

An alternative to the one-population Volterra model in Eq. (1) could be a generalization to a two-population Volterra model where both excitatory and inhibitory population firing rates are modeled explicitly. For the thalamocortical transformation this translates into including both an excitatory and an inhibitory layer-4 population, cf. Eqs. (14–15). With certain assumptions, however, this two-population model reduces to the one-population model in Eq. (1): with a *linear* inhibitory activation function, i.e., 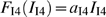, and no self-inhibition, i.e., 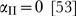 [53], we find from Eqs. (14–15) that
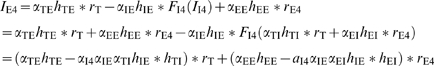
(43)


Then a term-by-term comparison shows that this expression has identical form as Eq. (1) provided the identifications
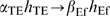
(44)

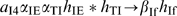
(45)

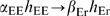
(46)

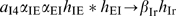
(47)are made.
